# Red Blood Cell‐Derived Exosomal miR‐93‐5p Promotes Lung Cancer Progression through PTEN Suppression

**DOI:** 10.1002/advs.202511940

**Published:** 2025-10-29

**Authors:** Ning Li, Pushpa Dhilipkannah, Van K Holden, Ashutosh Sachdeva, Feng Jiang

**Affiliations:** ^1^ Department of Pathology University of Maryland School of Medicine Baltimore MD 21021 USA; ^2^ Department of Medicine University of Maryland School of Medicine Baltimore MD 21021 USA; ^3^ BIOTARGET DX LLC 7193 Somerton Ct Hanover MD 21076 USA

**Keywords:** exosomal miRNAs, lung cancer, PTEN, red blood cells (RBCs), tumorigenesis

## Abstract

Red blood cells (RBCs), which are enucleated and mitochondria‐deficient, have traditionally been considered essential for gas exchange and systemic metabolic regulation. Here, an unrecognized role for RBCs in promoting lung cancer progression is identified. In a cohort of 226 lung cancer patients and 239 healthy controls, significantly elevated levels of miR‐93‐5p are observed in RBCs and RBC‐derived exosomes, but not in plasma, with higher levels correlating with advanced stage and poor prognosis. Functional assays demonstrate that RBC‐derived exosomal miR‐93‐5p is transferred to tumor cells, where it suppresses PTEN and enhances proliferation, migration, and invasion. Conversely, exosomes released by lung cancer cells deliver miR‐93‐5p to RBCs, thereby augmenting its abundance within RBCs. Inhibition of miR‐93‐5p or restoration of PTEN abrogates these effects. In both subcutaneous and orthotopic mouse models, RBC‐derived exosomal miR‐93‐5p accelerates tumor growth and reduces survival, whereas therapeutic delivery of antisense oligonucleotides targeting miR‐93‐5p suppresses tumor burden, reduces metastasis, and prolongs survival. Together, the novel function of RBCs as active participants in tumorigenesis is reported through exosomal transfer of oncogenic miR‐93‐5p, establishing a bidirectional tumor‐RBC‐tumor communication axis that promotes malignancy and offers new diagnostic and therapeutic opportunities in lung cancer.

## Graphical abstract

Red blood cell (RBC)‐derived exosomal miR‐93‐5p is elevated in lung cancer patients and correlates with poor outcomes. Exosomal miR‐93‐5p suppresses PTEN in cancer cells, enhancing proliferation, migration, and invasion. In mouse models, therapeutic inhibition with ASO‐miR‐93 5p reduces tumor growth, highlighting a bidirectional tumor‐RBC‐tumor axis and promising diagnostic and therapeutic targets.

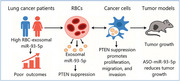

## 1 Introduction

Red blood cells (RBCs) are enucleated, mitochondria‐deficient cells conventionally recognized for delivering oxygen, removing carbon dioxide, and regulating body metabolic demands.^[^ ^1^ ^]^ RBCs have recently been uncovered to play multifaceted functions beyond their conventional role, including engaging in phagocytosis, antimicrobial defense, antigen recognition, and immune adherence.^[^ ^1^, ^2^, ^3^ ^]^An association between RBCs and cancer has been observed.^[^ ^3^, ^4^, ^5^, ^6^ ^]^ For example, RBCs can be present in the tumor microenvironment due to the leaky and disorganized vasculature commonly associated with malignant lesions.^[^ ^7^, ^8^ ^]^ Hypoxia within tumors, a condition caused by inadequate oxygen supply by RBCs, can lead to the selection of more aggressive cancer cell phenotypes, and increase resistance to chemotherapy and radiation therapy.^[^ ^9^ ^]^ Mature RBCs can acquire DNA from tumor tissues, and the presence of such mutated DNA has been proposed as a tool for early cancer detection.^[^ ^2^ ^]^ Previous studies have also explored the functional consequences of RBC‐tumor cell interactions, including the promotion of tumor cell invasiveness and the modulation of T‐cell activity through altered cytokine profiles.^[^ ^4^, ^10^ ^]^ However, the biological mechanisms by which RBCs contribute to tumorigenesis remain to be fully elucidated.

Exosomes, ranging in size from 20 to 200 nanometers, facilitate cellular communication, transporting various RNA species, such as microRNAs (miRNAs), along with DNA and proteins, from donor to recipient cells.^[^ ^11^ ^]^ These cargoes contribute to diverse physiological functions and are involved in promoting cancer progression and metastasis.^[^ ^11^, ^12^, ^13^, ^14^ ^]^ For instance, exosomes can alter the tumor microenvironment by facilitating angiogenesis in eluding the immune response.^[^ ^11^ ^]^ Additionally, exosomes can modulate the behavior of distant cells and facilitate the preparation of metastatic niches by transferring a diverse array of molecules.^[^ ^11^ ^]^ miRNAs play a crucial role in regulating gene expression, primarily at the post‐transcriptional level.^[^ ^15^ ^]^ miRNAs can function as oncomiRs or tumor suppressors by influencing key pathways involved in cell growth, cell death, and spread, thereby driving tumorigenesis.^[^ ^16^ ^]^ Furthermore, miRNAs within exosomes derived from cancer cells are pivotal in regulating processes related to malignancy.^[^ ^17^ ^]^ By transferring miRNAs between cells, exosomes can modulate gene expression in recipient cells, affecting cell proliferation, survival, and migration.^[^ ^18^ ^]^ Additionally, exosomal miRNAs from nucleated cells like fibroblasts, endothelial cells, mesenchymal stem cells, and immune cells can suppress tumor suppressors or enhance oncogenes, promoting tumor growth and chemotherapy resistance.^[^ ^19^ ^]^ RBCs are unexpectedly enriched in miRNAs.^[^ ^20^ ^]^ Given that RBCs lack a nucleus and transcriptional machinery, it was traditionally assumed that RBC‐derived miRNAs have limited biological relevance.^[^ ^21^ ^]^

Interestingly, our recent analysis of 133 blood samples identified a distinct miRNA signature in RBCs, prominently featuring miR‐93‐5p, which is closely associated with lung cancer.^[^ ^22^ ^]^ Notably, miR‐93‐5p is suggested as an oncomiRNA that plays a critical role in promoting cancer progression.^[^ ^23^, ^24^ ^]^ Our current study focuses on exploring the role of RBC‐derived miRNAs in the process of tumorigenesis, specifically targeting lung cancer, the leading cause of cancer mortality.^[^ ^25^ ^]^ Our study reveals that RBCs act as active contributors of oncogenic signaling through a novel bidirectional tumor‐RBC‐tumor communication axis mediated by exosomal miR‐93‐5p, thereby providing potential biomarkers and therapeutic targets in lung cancer.

## 2 Results

### 2.1 Clinical Characteristics of Study Participants

A total of 226 NSCLC patients and 239 cancer‐free smokers were enrolled in the study (Table S1, Supporting Information). The control group consisted of individuals with comparable smoking histories but no clinical or radiologic evidence of malignancy. Among NSCLC patients, the mean age was 68.4 years (SD = 12.2), with 34.1% female and 65.9% male, while the control group had a mean age of 66.2 years (SD = 11.5), with 36.0% female and 64.0% male. The racial distribution was comparable, with African Americans (AAs) and White Americans (WAs) accounting for 36.7% and 63.3% of NSCLC patients, respectively, and 36.0% and 64.0% of controls. All lung cancer cases were histologically confirmed as NSCLC, the predominant form of lung cancer,^[^ ^25^ ^]^ and were stratified by disease stage: 66 patients (29.2%) were classified as stage I, 126 (55.8%) as stage II, and 34 (15.0%) as stage III–IV. The lung cancer cases included the three major histological subtypes of NSCLC: 113 adenocarcinomas (AC, 50.0%), 90 squamous cell carcinomas (SCC, 39.8%), and 23 large cell carcinomas (LC, 10.2%). Median smoking pack‐years were 36 in the NSCLC group and 28 in controls. Complete clinical records and follow‐up data were available for all participants. The median overall survival (OS) for NSCLC patients was 20.5 months (range: 2–60 months; 95% CI: 17.5–23.6), with vital status confirmed through medical records, death certificates, or the Social Security Death Index. The procedural framework for patient inclusion and biospecimen analysis is summarized in Figure S1 (Supporting Information). This well‐characterized cohort provided a robust foundation for assessing the clinical relevance of RBC‐derived exosomal miRNAs in lung cancer pathogenesis.

### 2.2 Elevated miR‐93‐5p in RBCs and Exosomes Correlates with Advanced Tumor Stage and Poor Prognosis in Lung Cancer

**Figure 1:**
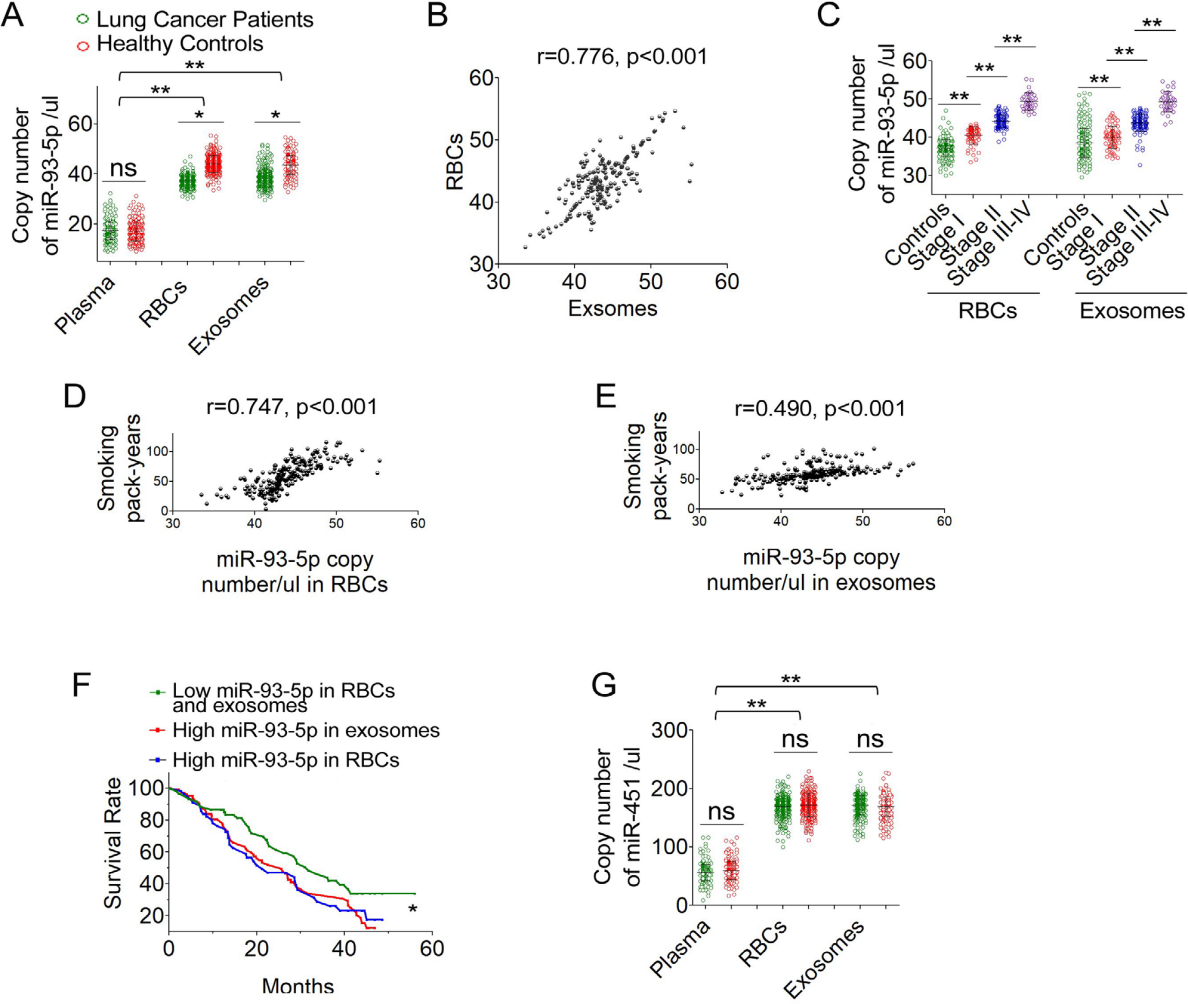
ddPCR analysis of miR‐93‐5p and miR‐451 in lung cancer patients and healthy controls. A) Expression levels of miR‐93‐5p were higher in RBCs and RBC‐derived exosomes of lung cancer patients (n = 226) compared to cancer‐free smokers (n = 239). Data are presented as mean ± SD. Unpaired two‐tailed Student's t‐tests were used (^*^ *p* < 0.05; ^**^ *p* < 0.01). Normality and variance equality were confirmed by the Shapiro–Wilk and Levene's tests. B) Positive correlation between miR‐93‐5p levels in RBCs and their exosomes (Pearson's correlation, r = 0.776, *p* < 0.001; n = 226). C) Stage‐related differences in RBC and exosomal miR‐93‐5p expression, with higher levels in stage II and further increases in stages III–IV. One‐way ANOVA with post hoc Tukey's test was applied (^*^ *p* < 0.05; n = 226). D–E) Correlations between RBC D) or RBC‐exosomal E) miR‐93‐5p and smoking pack‐years (r = 0.747 and 0.490; *p* < 0.001; Pearson correlation, n = 226). F) Kaplan–Meier analysis showed reduced survival in patients with high RBC‐ or exosomal‐miR‐93‐5p (P = 0.036; log‐rank test). G) miR‐451 levels were higher in RBCs and exosomes vs plasma in both groups, but did not differ between patients and controls. One‐way ANOVA with Tukey's post hoc test was applied (^*^ *p* < 0.05; ^*^ ^*^ *p* < 0.01; ns = not significant; n = 226 + 239).

Our previous research, conducted in a cohort comprising 68 lung cancer patients and 65 cancer‐free smokers, demonstrated that elevated miR‐93‐5p levels in RBCs are strongly associated with the presence of lung cancer.^[^ ^22^ ^]^ To investigate the association of RBC‐miR‐93‐5p with clinical outcomes, we used droplet digital PCR (ddPCR) to quantify miR‐93‐5p and miR‐451 (the housekeeper miRNA of RBCs) in RBCs, their corresponding exosomes, and plasma samples from the 226 lung cancer patients and 239 healthy controls who are cancer‐free smokers (Table S1, Supporting Information). To ensure sample purity and minimize contamination from blood cell lysis, plasma samples were processed within 2 h of collection using a double‐spin centrifugation protocol.^[^ ^26^, ^27^, ^28^, ^29^, ^30^, ^31^ ^]^ To ensure reproducible and high‐purity isolation of plasma‐derived exosomes, we followed a standardized protocol aligned with the Minimal Information for Studies of Extracellular Vesicles (MISEV) guidelines.^[^ ^31^ ^]^ Isolated exosomes were validated by nanoparticle tracking analysis (NTA) and transmission electron microscopy (TEM) to confirm size distribution, particle concentration, and morphological integrity.^[^ ^26^, ^27^, ^28^, ^29^, ^30^, ^31^ ^]^ Overall, miR‐93‐5p levels in RBCs and their exosomes were significantly higher than those in plasma, regardless of disease status (**Figure** **1**A). Interestingly, in patients with lung cancer, miR‐93‐5p levels were considerably elevated in both RBCs and their exosomes compared to controls (all *p* < 0.01) (Figure 1A). Furthermore, a strong correlation was found between miR‐93‐5p levels in RBCs and their exosomes, specifically in lung cancer patients (r = 0.776, p < 0.001) (Figure 1B). Moreover, elevated levels of miR‐93‐5p in RBCs and the exosomes were significantly associated with advanced lung cancer stages and smoking history (all *p* < 0.05) (Figure 1C–E) (Tables S3 and S4, Supporting Information). In multivariable Cox regression analysis adjusted for tumor stage and smoking history, RBC and exosomal miR‐93‐5p remained significant predictors of overall survival (Figure 1F; Table S5, Supporting Information), whereas plasma miR‐93‐5p showed a nonsignificant trend. Tumor stage was a strong independent predictor, and smoking contributed modestly. miR‐451, a housekeeping miRNA highly expressed in RBCs, was abundantly enriched in both RBCs and their exosomes but not in plasma. However, its expression levels did not differ significantly between patient and control groups and were not associated with outcome (all *p* > 0.05) (Figure 1G; Tables S5 and S6, Supporting Information).

### 2.3 RBC‐Derived miRNAs can be Transferred to Lung Cancer Cells Predominantly via Exosomal Pathways

**Figure 2:**
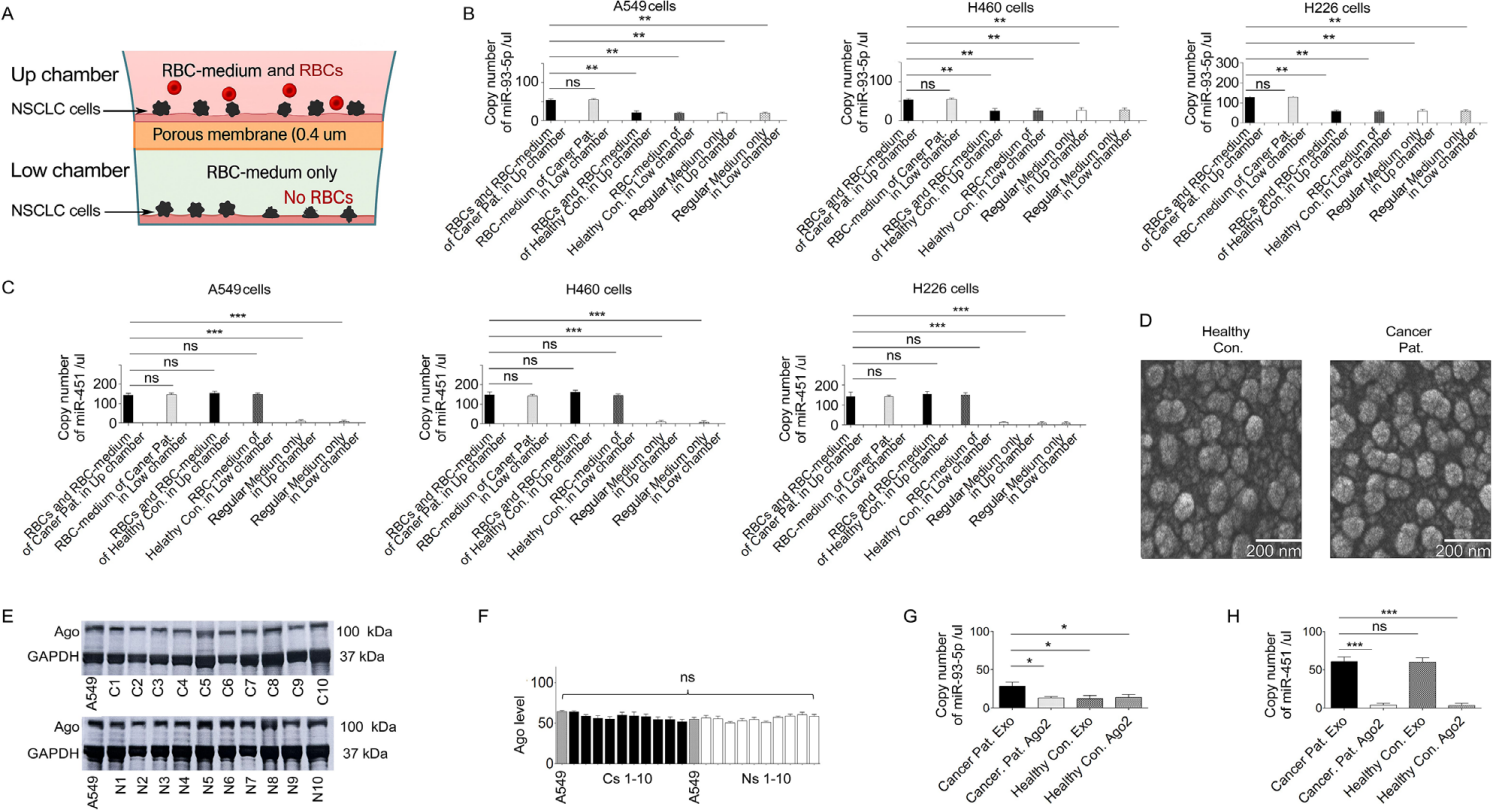
RBC‐Derived miRNA Transfer to Lung Cancer Cells via Exosomes. A) Schematic of a dual‐chamber Transwell system with a 0.4 µm membrane. RBCs and RBC‐conditioned medium (RBC‐medium) are maintained in the up chamber, while only the RBC‐medium passes to the low chamber, excluding intact RBCs. Lung cancer cells in the up chamber are exposed to both RBCs and RBC‐medium, whereas cells in the low chamber are exposed only to RBC‐medium. B) Expression levels of miR‐93‐5p in NSCLC cells after exposure to RBCs from cancer patients (Can. Pat.) and healthy controls (Healthy Con.), analyzed from the up chamber, low chamber, and regular medium as a negative control, indicating that RBC‐derived miRNAs are primarily transferred via the RBC‐ medium without direct contact. C) Relative expression levels of miR‐451 under the same conditions, supporting the same conclusion. (One‐way ANOVA) (Normality was assessed using the Shapiro–Wilk test, and homogeneity of variance was verified using Levene's test prior to ANOVA). D) Transmission electron microscopy (TEM) images of exosomes isolated from RBC‐conditioned medium of a healthy control (upper panel) and a lung cancer patient (lower panel). Both images were captured at ×100 000 magnification, and the scale bar represents 200 nm. Exosomes appear as round, membrane‐bound vesicles with a typical diameter of 30–150 nm. These images confirm the successful isolation and morphology of exosomes from both groups, with increased vesicle abundance observed in the cancer patient sample. Annotations have been added to clearly identify the exosome source and distinguish the upper and lower panels. E) Western blot analysis confirming the presence of Ago2 protein in exosome fractions from RBC‐medium of ten lung cancer patines (Cs) and ten normal healthy controls (Ns) with GAPDH used as a loading control. The A549 NSCLC cell line was included as a control. F) Relative Ago protein expression was quantified and presented as a bar graph, with levels normalized to GAPDH (set as 100%). There is no difference in Ago expression between tumor and normal lung tissues (p = 0.356). G) miR‐93‐5p levels were significantly higher in both exosomes and Ago2‐immunoprecipitated complexes from cancer patients compared to healthy controls. H) miR‐451 levels were significantly higher in exosomes from cancer patients, but showed no significant difference in Ago2 complexes between the two groups. All tests assumed normal distribution and equal variances, confirmed prior to analysis using the Shapiro–Wilk and Levene's tests, respectively. All assays were conducted using a minimum of three independent biological replicates per group. Please note: ^*^ *p* < 0.05; ^*^ ^*^ *p* < 0.01; ^**^ ^*^ *p* < 0.001; ns, not significant.

To investigate whether RBC‐derived miRNAs are transferred to lung cancer cells, we collected RBCs from 26 lung cancer patients with high miR‐93‐5p levels and 26 healthy controls with low levels (Table S7, Supporting Information). We also obtained human NSCLC cell lines representing the three major histological subtypes: A5492 (AC), H226 (SCC), and H460 (LCC). We then designed a dual‐chamber Transwell co‐culture system equipped with a 0.4 µm porous membrane that separates the upper and lower chambers (**Figure** **2**A). The pore size was selected to prevent RBCs (which are ≈6–8 µm in diameter) from passing through the membrane, while permitting the diffusion of small extracellular vesicles (EVs), such as exosomes (≈30–150 nm), and soluble factors. In this setup, RBCs are added only in the upper chamber, ensuring that any molecular communication to the lower chamber occurs via secreted components (referred to as RBC‐conditioned medium or RBC‐medium). NSCLC cells are cultured in both chambers: those in the upper chamber are directly exposed to both RBCs and RBC‐medium, while cells in the lower chamber are exposed exclusively to RBC‐medium. This shared‐medium design allows for the separation of direct RBC effects from those mediated solely by secreted factors. To further minimize potential confounding from cancer cell‐derived miRNAs, the co‐culture duration was restricted to 24 h or less, thereby limiting the accumulation of miRNAs secreted by cancer cells into the shared medium. Under these conditions, miRNA levels in the medium predominantly reflected RBC‐derived contributions. NSCLC cells in both chambers after 24 h of incubation were subsequently harvested for RNA extraction to evaluate intracellular miR‐93‐5p uptake. As shown in Figure 2B, NSCLC cells in the upper chamber incubated with both RBCs and RBC‐medium from lung cancer patients exhibited miR‐93‐5p levels comparable to those incubated with RBC‐medium alone in the lower chamber (all p > 0.05). Furthermore, NSCLC cells in the upper chamber incubated with both RBCs and RBC‐medium from lung cancer patients exhibited significantly higher miR‐93‐5p levels compared to those cultured with RBCs and conditioned medium from healthy controls (*p* < 0.001, Figure 2B). Similarly, NSCLC cells in the lower chamber showed increased miR‐93‐5p levels when exposed to RBC‐medium from lung cancer patients vs healthy controls (*p* < 0.001, Figure 2B). In contrast, NSCLC cells cultured with regular medium in both up and low chambers exhibited very low miR‐93‐5p levels (Figure 2B). For miR‐451, a housekeeping gene, NSCLC cells in the upper chamber incubated with both RBCs and RBC‐conditioned medium from lung cancer patients showed miRNA levels comparable to those incubated with RBC‐conditioned medium alone in the lower chamber (all *p*> 0.05, Figure 2C).

miR‐451 levels were also similar in NSCLC cells treated with RBCs and RBC‐medium from lung cancer patients vs those treated with RBCs and conditioned medium from healthy controls in both chambers (*p* > 0.05, Figure 2C). NSCLC cells cultured in regular medium in both chambers exhibited very low miR‐451 expression (*p* < 0.01) (Figure 2C). Collectively, the comparable intracellular miRNA levels detected in NSCLC cells cultured in the upper chamber (exposed to both RBCs and RBC‐conditioned medium) and in the lower chamber (exposed only to RBC‐conditioned medium) indicate that RBC‐derived miRNAs are delivered to cancer cells via secreted factors present in the shared medium (RBC‐conditioned medium).

Given the lack of nucleus‐based machinery in RBCs, it is proposed that exosomes and Argonaute 2 (Ago2) function as primary transporters of molecules crucial for facilitating communication from RBCs to other cells.^[^ ^21^, ^32^ ^]^ To further explore the mechanism through which RBC‐derived miR‐93‐5p is transferred to lung cancer cells, we collected RBCs from 10 lung cancer patients and 10 healthy controls, cultured them, and isolated exosomes (Figure 2D) and Ago2‐associated immunoprecipitation (IP) products from the RBC‐medium. Western blot analysis confirmed the presence of Ago2 protein in exosome fractions from RBC‐conditioned medium of both cancer patients and healthy controls, with no significant difference in Ago2 expression between the two groups (Figure 2E,F). Subsequently, RNA was extracted from these exosomes and Ago2 IPs for miRNA quantification. The exosomes showed significantly higher levels of miR‐93‐5p compared to very low levels in the Ago2 IPs from the same lung cancer patients (Figure 2G). Similarly, both exosomes and Ago2 IPs from healthy controls exhibited low miR‐93‐5p levels. Additionally, while exosomes demonstrated significantly elevated levels of miR‐451, this miRNA was notably low in the matched IPs from both lung cancer patients and healthy controls (Figure 2H). Collectively, the predominance of miRNAs in exosomes, rather than in Ago2 complexes, confirms that exosomes derived from RBCs are the principal transporters of these miRNAs, playing a crucial role in mediating intercellular communication to cancer cells.

**Figure 3:**
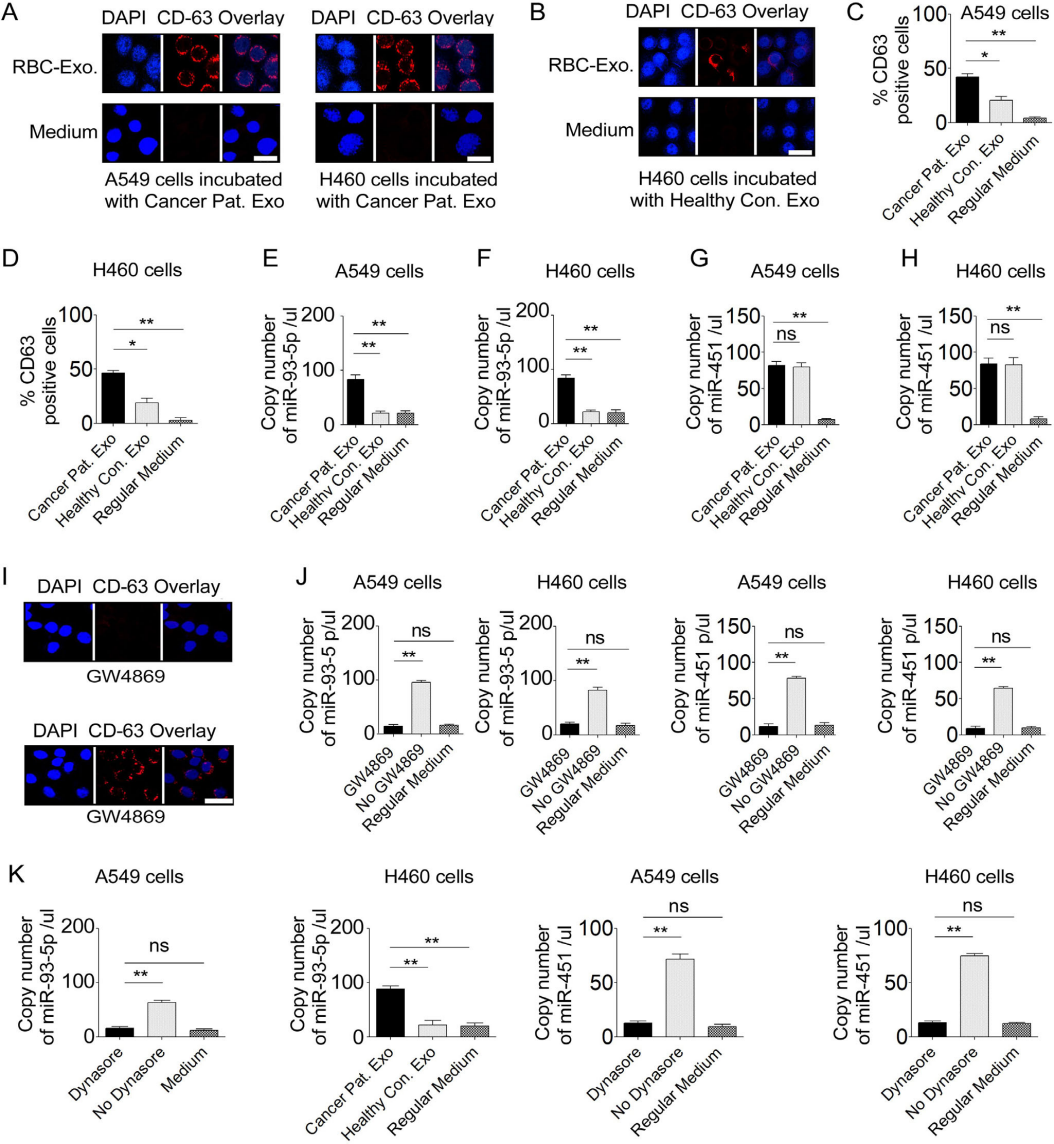
RBC‐Derived Exosomal Transfer of miR‐93‐5p and miR‐451 to Lung Cancer Cells. A) Fluorescent microscopy images displaying uptake of CD63‐positive (red) RBC‐derived exosomes from lung cancer patients by NSCLC cells, with nuclei stained blue (DAPI). Scale bar: 20 µm. B) Similar uptake of CD63‐positive (red) RBC‐derived exosomes from healthy controls by NSCLC cells. C,D) Quantitative analysis demonstrating a significant increase in the uptake of CD63‐positive RBC‐derived exosomes from cancer patients compared to healthy controls, with no uptake observed from basal medium (control medium). n = 3 independent experiments; data = mean ± SD, ^*^ *p* < 0.01, ^*^ ^*^ *p* < 0.001. E–H) A549 and H460 cancer cells showed increased levels of both miR‐93‐5p and miR‐451 after incubation with exosomes from cancer patients, but only miR‐451 was elevated following exposure to exosomes from cancer‐free controls (n = 3). Data = mean ± SD; ^*^ ^*^ *p* < 0.001. I) Fluorescent images show reduced CD63 staining in A549 cells treated with the exosome release inhibitor GW4869 compared to untreated cells, indicating decreased exosome secretion. Scale bar: 20 µm. J) Impact of GW4869 treatment on miR‐93‐5p and miR‐451 levels in A549 and H460 cells, displaying significantly reduced miRNA levels in treated cells compared to controls (n = 3; Student's t‐test). ns = not significant; ^*^ ^*^ *p* < 0.001. K) Impact of the exosome uptake inhibitor Dynasore on miR‐93‐5p and miR‐451 levels in A549 and H460 cells, displaying significantly reduced miRNA levels in treated cells compared to controls. ns = not significant; ^*^ ^*^ *p* < 0.001. Statistical analyses were performed using two‐tailed Student's t‐tests. Normality was confirmed using the Shapiro–Wilk test, and equality of variance was assessed using Levene's test. Data are presented as mean ± SD. Three human NSCLC cell lines were used in these experiments, consistently demonstrating similar results; data for A549 and H460 cell lines are presented. All assays were conducted using a minimum of three independent biological replicates per group (n ≥ 3).

To further confirm these findings, we evaluated the interaction between RBC‐derived exosomes and NSCLC cells by performing immunofluorescence staining using an anti‐CD63 antibody, a widely accepted marker for exosomes.^[^ ^33^ ^]^ Following a 24 h incubation, CD63‐positive puncta were detected in NSCLC cells treated with exosomes from both lung cancer patients and healthy controls, indicating cellular association through binding and/or uptake (**Figure** **3**A,B).

However, the frequency of CD63‐positive cells was significantly higher in those exposed to patient‐derived exosomes compared to controls (Figures 3C,D, all *p* < 0.01) (Figure S2A, Supporting Information), suggesting enhanced exosome interaction. Consistently, NSCLC cells treated with cancer patient‐derived exosomes exhibited elevated levels of both miR‐93‐5p and miR‐451, whereas cells exposed to exosomes from healthy controls showed increased levels of miR‐451 only, likely reflecting the lower abundance of miR‐93‐5p in exosomes from non‐cancer donors (Figure 3E–H) (Figure S2B, Supporting Information).

To further validate the above observations, we used GW4869, a well‐characterized inhibitor of exosome release, to treat RBCs. The resulting RBC‐medium, collected from both GW4869‐treated and untreated RBCs, was applied to NSCLC cells for 24 h. Immunofluorescence staining revealed a lack of CD63‐positive exosomes in NSCLC cells treated with medium from GW4869‐treated RBCs (Figure 3I). Correspondingly, these cells exhibited undetectable levels of miR‐93‐5p and miR‐451 (Figure 3J) (Figure S2C, Supporting Information), indicating that inhibition of exosome release from RBCs effectively blocks miRNA transfer to NSCLC cells. To further confirm that miRNA delivery is mediated by exosome uptake, RBC‐medium was co‐incubated with NSCLC cells in the presence or absence of Dynasore, an inhibitor of endocytic internalization. After 24 h, cells treated with Dynasore failed to uptake RBC‐derived exosomes and showed no increase in intracellular miR‐93‐5p or miR‐451 levels. In contrast, cells incubated without the inhibitor exhibited significant uptake of RBC‐derived exosomes and elevated levels of both miRNAs (Figure 3K) (Figure S2D, Supporting Information). Collectively, these results further support the role of RBC‐derived exosomes as key vehicles mediating the transfer of miRNAs to NSCLC cells.

### 2.4 The RBC‐Derived miR‐93‐5p Promotes Cell Proliferation, Invasiveness, and Migration

We further investigated whether the transfer of RBC‐derived miR‐93‐5p to NSCLC cells could enhance their tumorigenic properties. RBC‐derived exosomes were collected from 16 lung cancer patients and eight healthy controls, respectively. The lung cancer patients included eight with high levels of miR‐93‐5p and eight with low levels of miR‐93‐5p in their RBCs and exosomes.

**Figure 4:**
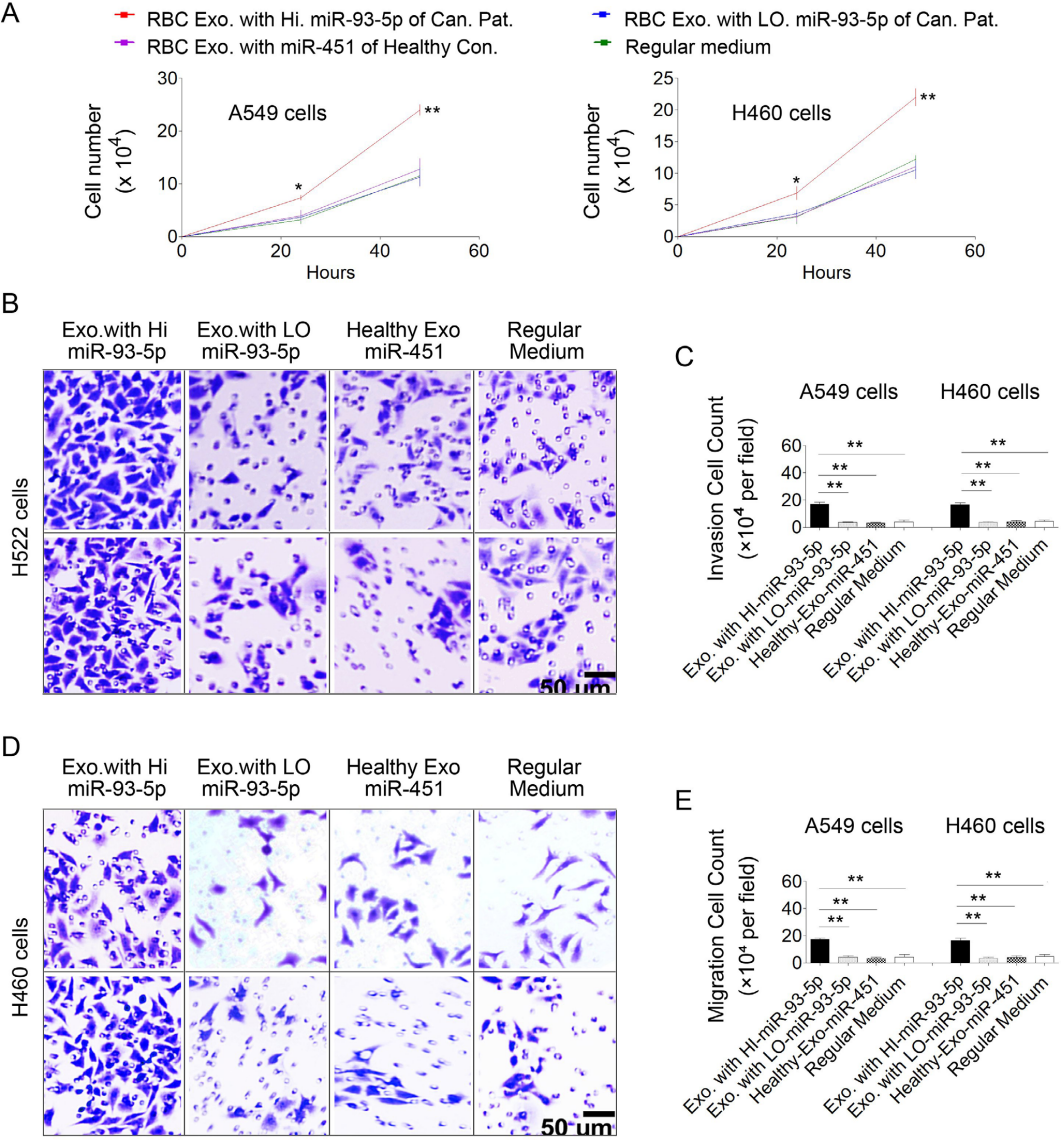
Effects of RBC‐derived Exosomal miR‐93‐5p on Tumorigenicity of NSCLC Cells. A) Cancer cells incubated with RBC‐exosomes from lung cancer patients with high levels of miR‐93‐5p show increased proliferation compared to those incubated with RBC‐exosomes from lung cancer patients with low miR‐93‐5p and healthy controls. B) Invasion assays for cancer cells treated similarly demonstrate enhanced invasive capabilities with RBC‐exosomal miR‐93‐5p from lung cancer patients. C) Bars represent the number of invasive cells per field, confirming increased invasion with high miR‐93‐5p exosomes. D) Migration assays for cancer cells treated with the same groups of exosomes, showing increased migration capabilities when treated with exosomes from cancer patients with high miR‐93‐5p. E) Quantification of migration assays shown in (D). Bars represent the number of migrated cells per field, demonstrating enhanced migration with high miR‐93‐5p exosomal treatment. Data are mean ± SD from n ≥ 3 biological replicates. Statistical comparisons by Student's t‐test (^*^ *p* < 0.05; ^*^ ^*^ *p* < 0.01). Three NSCLC cell lines were used in the experiments, all of which displayed the same results. All assays were conducted using a minimum of three independent biological replicates per group (n ≥ 3).

NSCLC cell lines (A549, H460, and H226) and normal bronchial epithelial cells (NHBE) were treated with RBC‐derived exosomes from lung cancer patients and healthy controls, respectively, and subsequently analyzed for miR‐93‐5p uptake and associated functional phenotypes. Compared to NSCLC cells treated with exosomes from healthy donors or patients with low miR‐93‐5p, those exposed to high‐miR‐93‐5p exosomes showed significantly increased intracellular miR‐93‐5p levels and pronounced increases in cell proliferation (**Figure** **4**A), invasion (Figure 4B,C), and migration (Figure 4D,E). Importantly, incubation with RBC‐derived exosomes enriched in miR‐451, a housekeeping miRNA, did not promote tumorigenic behaviors in any cell line, confirming the specificity of the oncogenic effects of miR‐93‐5p. In contrast, normal bronchial epithelial cells (NHBE) did not show any increase in malignant behavior upon treatment (Figure S3, Supporting Information). Therefore, the transfer of miR‐93‐5p, a lung cancer‐associated RBC‐miRNA, to NSCLC cells may enhance malignant properties, in contrast to the effect of transferring RBCs' housekeeping miRNA, miR‐451.

### 2.5 The Transferred RBC‐Derived miR‐93‐5p Targets the PTEN Gene in Cancer Cells and Promotes Tumorigenesis

**Figure 5:**
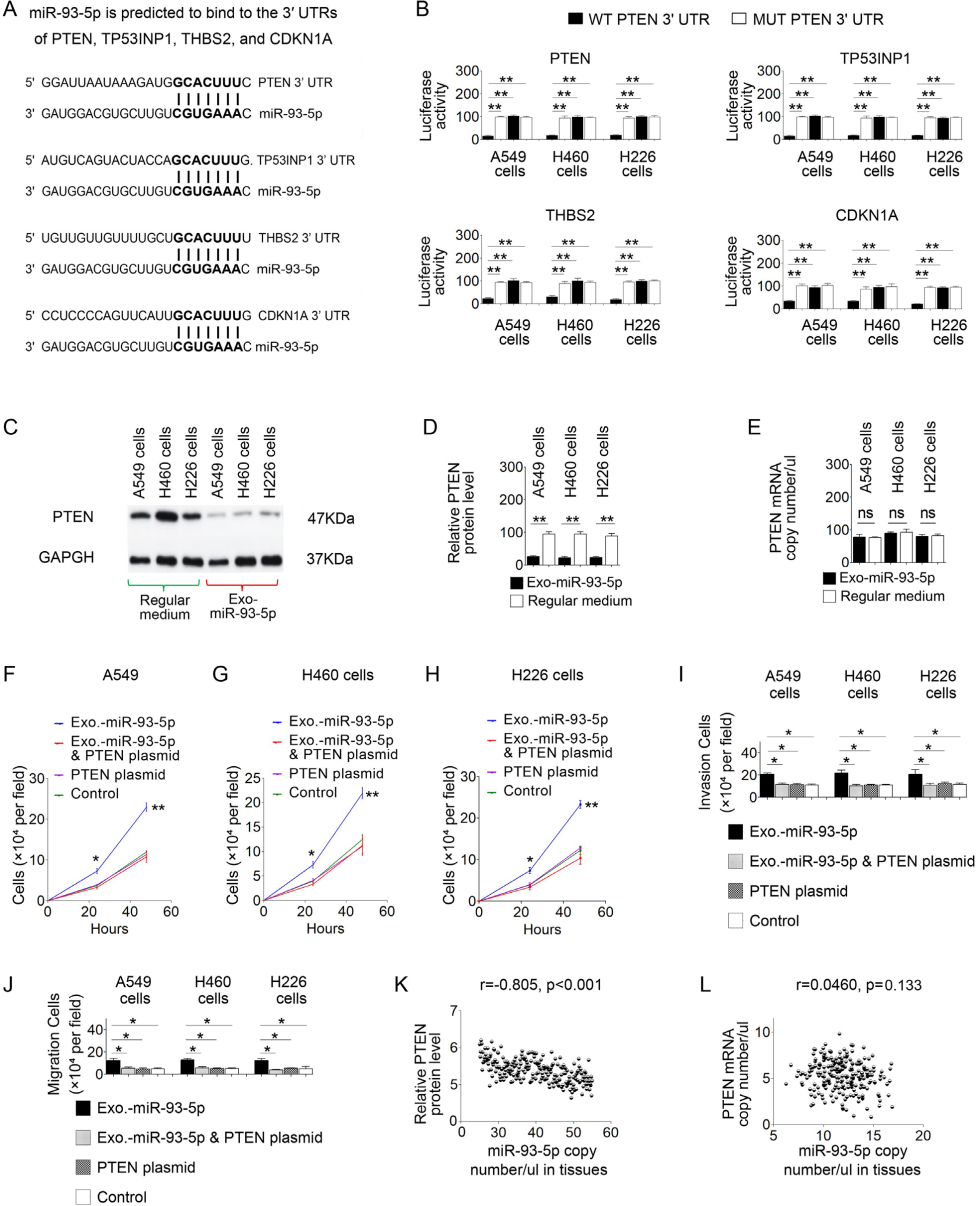
Impact of RBC‐Exosomal miR‐93‐5p on PTEN Regulation and Tumorigenicity in Lung Cancer Cells. A) Sequence alignment illustrates the predicted binding sites of miR‐93‐5p on the 3′UTRs of PTEN, TP53INP1, THBS2, and CDKN1A, highlighting wild‐type (WT) and mutant (MUT) sequences. B) Luciferase reporter assays show significantly reduced luciferase activity in lung cancer cells co‐transfected with WT 3′UTRs of these genes, confirming direct targeting by miR‐93‐5p. C) Western blot analysis indicates that RBC‐derived exosomal miR‐93‐5p downregulates PTEN protein levels in lung cancer cells, with GAPDH as a loading control. D,E) Quantification of PTEN protein and mRNA levels in A549 and H460 cells reveals a significant reduction in PTEN protein expression, whereas PTEN mRNA levels remain unchanged. F–H) Cell proliferation assays demonstrate that exosomal miR‐93‐5p enhances lung cancer cell growth, while restoring PTEN expression using a PTEN expression plasmid reduces proliferation. I,J) Invasion and migration assays show that exosomal miR‐93‐5p promotes invasion and migration, while PTEN restoration counteracts these effects. K) Analysis of 226 NSCLC tumor tissues revealed a strong inverse correlation between miR‐93‐5p levels and PTEN protein expression in patients with wild‐type PTEN, as determined by Pearson correlation analysis (*p* < 0.01). L) no correlation is observed between miR‐93‐5p levels and PTEN mRNA expression (p = 0.133). All statistical tests assumed normality of data distribution (Shapiro–Wilk test, *p* > 0.05) and equality of variances (Levene's test, *p* > 0.05) prior to applying Student's t‐tests or Pearson correlation analyses. Data are presented as mean ± SD. ns = not significant; ^*^ *p* < 0.05; ^*^ ^*^ *p* < 0.01. All assays were conducted using a minimum of three independent biological replicates per group (n ≥ 3).

To investigate the mechanism by which RBC‐derived miR‐93‐5p promotes tumorigenesis, we used bioinformatic prediction tools to identify potential gene targets of this miRNA. The analysis identified miR‐93‐5p binding sites within the 3′‐UTRs of several key tumor‐associated genes, including PTEN, CDKN1A (p21), TP53INP1, and THBS2 (**Figure** **5**A). Luciferase reporter assays confirmed the prediction for these genes by showing decreased luciferase activity in cells co‐transfected with wild‐type (WT) ‐3′UTR and a miR‐93‐5p mimic, while no such effect was observed in cells transfected with mutant (MUT) constructs (Figure 5B). The results imply that miR‐93‐5p may directly bind to the mRNA of these genes, leading to their degradation or translational inhibition.

Among the predicted targets of miR‐93‐5p, PTEN is a tumor suppressor of particular importance in lung cancer, where it is frequently inactivated or downregulated. We therefore focused on PTEN to assess the functional impact of miR‐93‐5p in lung cancer. To determine whether the oncogenic effects of miR‐93‐5p depend on PTEN status, we first analyzed six NSCLC cell lines representing the major histological subtypes, AC, SCC, and LC, with defined PTEN genotypes (Table S2, Supporting Information). The PTEN wild‐type group included A549 (AC), H226 (SCC), and H460 (LC), while the PTEN‐altered group (mutated or null) consisted of H23 (AC), H520 (SCC), and H810 (LC). Normal bronchial epithelial cells (NHBE and BEAS‐2B) were included as non‐malignant controls. In PTEN wild‐type NSCLC cell lines, treatment with RBC‐derived exosomes enriched in miR‐93‐5p led to a marked reduction in PTEN protein expression, without changes in PTEN mRNA levels (Figure 5C–E), indicating post‐transcriptional suppression. In contrast, NSCLC cells with PTEN mutations or deletions (H23, H520, and H810) exhibited minimal phenotypic changes upon exposure to the same exosomes (Figure S4, Supporting Information), consistent with the loss of functional PTEN as a target of miR‐93‐5p. Similarly, normal bronchial epithelial cells did not exhibit oncogenic transformation upon treatment with miR‐93‐5p‐rich exosomes (Figure S4, Supporting Information).

To validate the oncogenic role of miR‐93‐5p via PTEN suppression, we ectopically expressed PTEN in PTEN wild‐type NSCLC cells using a pcDNA3.1‐PTEN construct lacking the 3′ UTR. Transfection efficiency was confirmed by Western blot and fluorescence co‐reporter assays. Ectopic expression of 3′UTR‐less PTEN effectively elevated PTEN protein levels and counteracted miR‐93‐5p‐induced malignant phenotypes, including increased proliferation and enhanced cell motility (Figure 5F–J), thereby supporting a direct mechanistic link. Additionally, knockdown of endogenous miR‐93‐5p using antisense oligonucleotides (ASO‐miR‐93‐5p), validated by ddPCR and target de‐repression assays, significantly attenuated the tumor‐promoting effects of RBC‐derived exosomes (Figure S5, Supporting Information). Collectively, these findings demonstrate that RBC‐derived miR‐93‐5p promotes tumorigenic behavior in NSCLC cells via post‐transcriptional suppression of PTEN, and that these effects are contingent on the presence of functional PTEN protein.

### 2.6 RBC miR‐93‐5p Levels are Inversely Associated with PTEN Protein Expression in Human Tumors Harboring Wild‐Type PTEN

To explore whether miR‐93‐5p‐mediated PTEN inactivation is relevant in clinical settings, we analyzed tumor tissues from 226 NSCLC patients using next‐generation sequencing (NGS). Targeted sequencing of PTEN hotspot regions, primarily exons 5–8, revealed genomic alterations (mutations or deletions) in 29 cases (12.7%), consistent with previous reports.^[^ ^34^ ^]^ Stratification by disease stage showed an increasing frequency of PTEN alterations: 5.6% in Stage I, 7.9% in Stage II, and 11.1% in Stage III–IV (Table S8, Supporting Information), suggesting that PTEN inactivation may contribute to tumor progression. By histological subtype, PTEN mutations were most frequent in AC (5.8%) and SCC (5.0%), followed by LC (2.7%) (Table S9, Supporting Information).

We further quantified PTEN protein levels in tumor tissues using enzyme‐linked immunosorbent assay (ELISA) and PTEN mRNA expression using ddPCR, and compared these with matched RBC‐derived miR‐93‐5p levels to assess potential regulatory relationships. In tumors harboring wild‐type PTEN, miR‐93‐5p levels were significantly inversely correlated with PTEN protein expression (*p* < 0.05), while no correlation was observed with PTEN mRNA levels (*p* = 0.133) (Figure 5K,L), supporting a post‐transcriptional mode of regulation. In contrast, no significant correlation was observed in tumors with PTEN mutations or deletions (*p* > 0.05) (Figure S6, Supporting Information), suggesting that genetic inactivation of PTEN renders tumor cells unresponsive to miR‐93‐5p–mediated suppression. These findings, consistent with the in vitro functional assays, confirm that miR‐93‐5p primarily downregulates PTEN protein levels through post‐transcriptional mechanisms. Moreover, miR‐93‐5p promotes oncogenic phenotypes only in the context of functionally intact PTEN, and its tumor‐promoting effects are attenuated in cases where PTEN is inactivated. Taken together, our results highlight the critical role of PTEN integrity in modulating the oncogenic activity of miR‐93‐5p in NSCLC.

### 2.7 RBC‐Derived miR‐93‐5p Enhances Lung Tumor Growth in Subcutaneous and Orthotopic Xenograft Models

**Figure 6:**
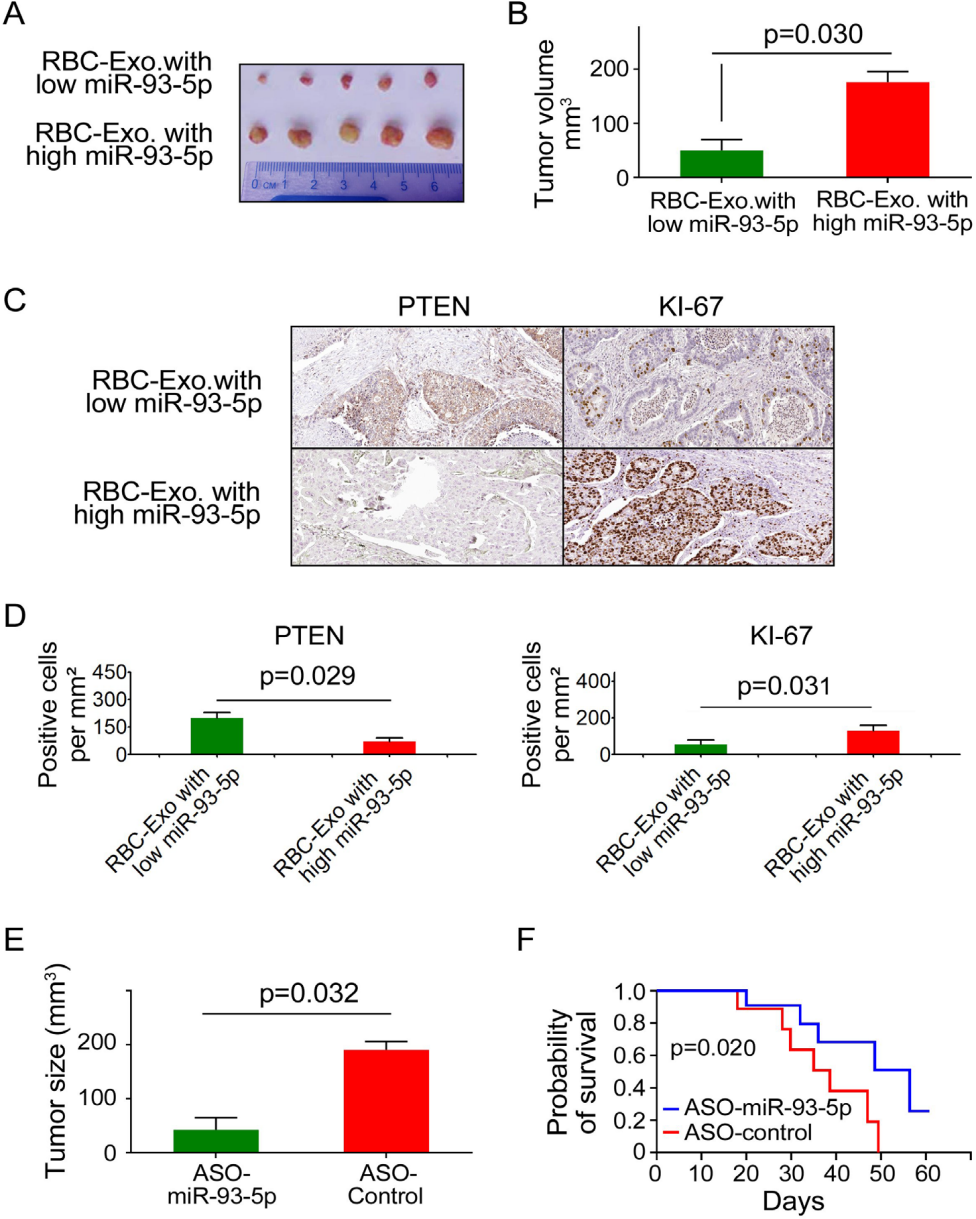
RBC‐Derived miR‐93‐5p Promotes Tumor Growth and Decreases Survival in Subcutaneous Lung Cancer Xenograft Models. A) Representative images of tumors harvested at week 4 from mice injected with RBC‐derived exosomes containing low or high levels of miR‐93‐5p show significantly larger tumors in the high miR‐93‐5p group. B) Tumor volume quantification confirmed this increase (Y‐axis indicates tumor volume in mm^3^, measured by caliper‐based ellipsoid calculation) (*p* = 0.030) (n = 8 per group). C) Immunohistochemical (IHC) staining of tumor sections revealed reduced PTEN expression and increased Ki‐67 levels in the high miR‐93‐5p group. D) Quantitative analysis confirmed a significant reduction in PTEN‐positive cells (*p* = 0.029) and an increase in Ki‐67‐positive cells (*p* = 0.031). E) In a separate experiment, tumor‐bearing mice treated with ASO‐miR‐93‐5p exhibited a significant reduction in tumor size compared to the ASO‐control group (*p* = 0.032). F) Kaplan–Meier survival analysis showed that ASO‐miR‐93‐5p‐treated mice had significantly prolonged survival compared to ASO‐controls (*p* = 0.020). Statistical analyses were performed using Student's t‐test for normally distributed data with approximately equal variances (confirmed by Shapiro–Wilk and Levene's tests). Survival curves were compared using the log‐rank (Mantel–Cox) test. Data are presented as mean ± SD.

The mice were randomly divided into two groups: Group 1 received subcutaneous injections of H460‐Luc cells treated with RBC‐derived exosomes expressing low levels of miR‐93‐5p from a healthy individual. Conversely, Group 2 was injected with H460‐Luc cells treated with RBC‐derived exosomes that exhibit high expression of miR‐93‐5p from lung cancer patients. By the end of the fourth week, tumors derived from the Group 2 were notably larger, with an average volume of 88.39±14.26 mm^3^, in contrast to the tumors in group 1, which averaged 25.75±15.48mm^3^ (p = 0.049) (**Figure** **6**A,B). These tumors demonstrated reduced PTEN expression and elevated KI‐67 levels when compared to tumors from the control group (all p < 0.05) (Figure 6C‐D). The results from the xenograft mouse model further support the crucial role of RBC‐derived miR‐93‐5p in promoting lung cancer development by targeting PTEN.

### 2.8 Effective Antitumor Effects of ASO‐miR‐93‐5p In Vivo

To explore the impact of inhibiting RBC‐derived miR‐93‐5p on tumor growth in vivo, mice were injected with H460‐Luc cells treated with RBC‐derived exosomes with high expression of miR‐93‐5p from lung cancer patients. Within 10 days, tumors had formed in all the mice injected with cancer cells. Subsequently, these mice were randomly divided into two groups. Group 1 received direct injections into the tumors with ASO‐miR‐93‐5p, while Group 2 received a control ASO, administered three times per week for three weeks. Tumors treated with ASO‐miR‐93‐5p exhibited a significantly larger reduction in volume compared to the control group (190.52±21.92 mm^3^ vs 42.39±31.82 mm^3^, p = 0.032) (Figure 6E) (Figure S7, Supporting Information). Furthermore, mice receiving ASO‐miR‐93‐5p exhibited a longer survival period than those treated with control ASO (p = 0.018) (Figure 6F). Thus, miR‐93‐5p inhibition could effectively reduce tumor growth in vivo, highlighting its potential as a therapeutic strategy.

Furthermore, we employed an orthotopic lung cancer model to evaluate the oncogenic function of RBC‐derived miR‐93‐5p and the therapeutic potential of its inhibition. Orthotopic lung tumors formed from H460 NSCLC cells treated with RBC‐derived exosomes containing high levels of miR‐93‐5p grew significantly faster than those formed from cells pretreated with low‐miR‐93‐5p exosomes. Postmortem tumor volume analysis confirmed markedly larger tumors in the high‐miR‐93‐5p group (*p* = 0.031) (Figure S8, Supporting Information). Furthermore, the orthotopic lung cancer model developed metastases primarily in the contralateral lung lobes and mediastinal lymph nodes, as confirmed by histological examination using H&E staining (Figures S9 and S10, Supporting Information), which revealed features consistent with a LC subtype of NSCLC, in agreement with the classification of the implanted H460 cells. Notably, ASO‐miR‐93‐5p therapy significantly reduced orthotopic lung tumor volumes as well as the number of metastatic lesions per mouse (all *p* < 0.05; Figures S8 and S10, Supporting Information). These results confirm and extend findings from the above subcutaneous xenograft model, reinforcing the tumor‐promoting role of RBC‐derived miR‐93‐5p and demonstrating that its inhibition not only suppresses primary tumor growth but also reduces metastatic potential in a microenvironment that more closely mimics human lung cancer.

### 2.9 Lung Cancer Cells Contribute as One of the Primary Sources of RBC‐Derived miR‐93‐5p Through Exosome Transfer

**Figure 7:**
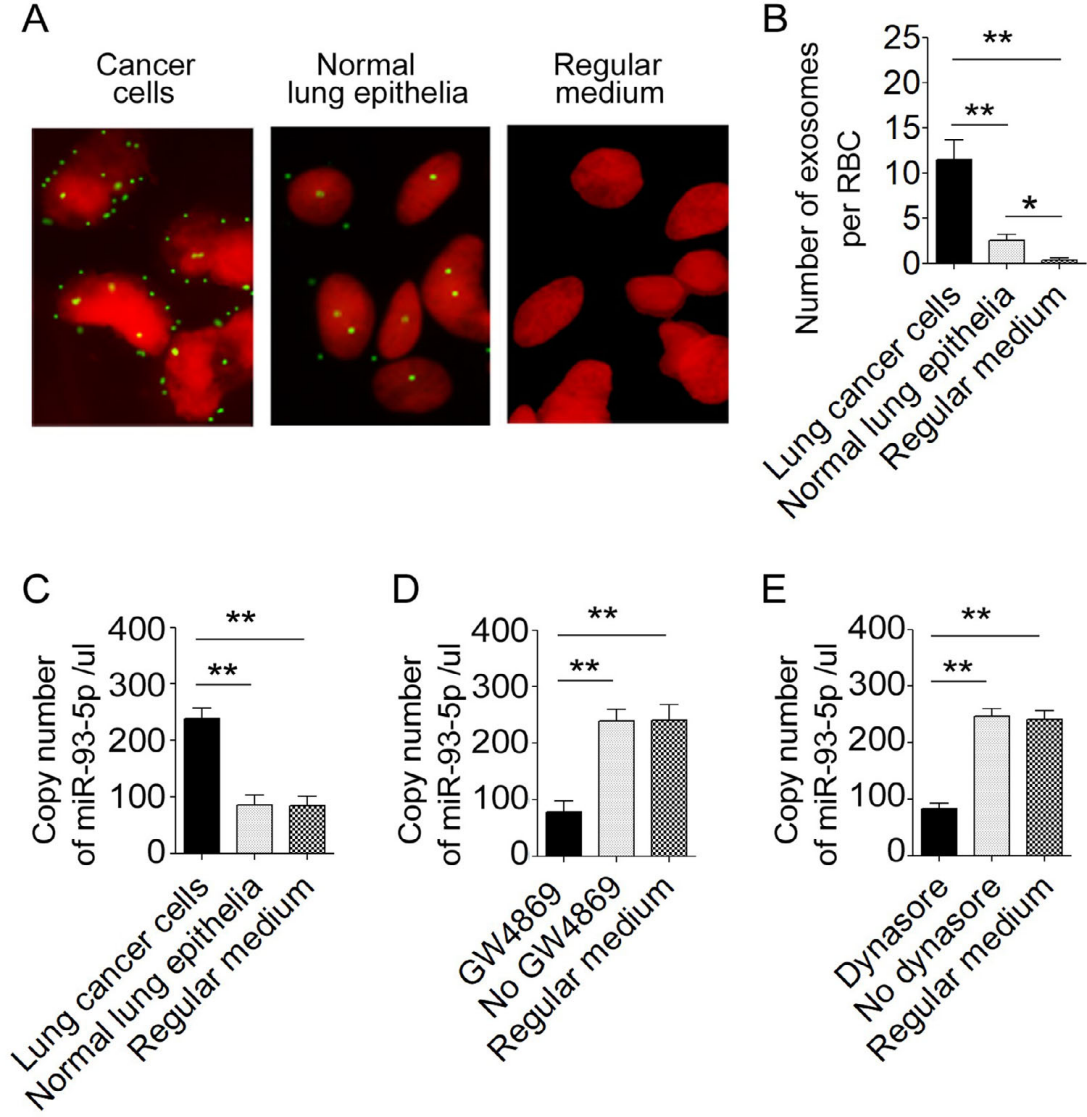
Lung cancer cell‐derived exosomes load miR‐93‐5p into RBCs. A) Representative images of RBCs (red) incubated with fluorescently labeled exosomes from lung cancer cells (H226), normal epithelial cells, or medium. Numerous exosomes (green) attach to RBCs in the cancer‐exosome condition, with fewer in normal epithelia and negligible in regular medium. B) Quantification of exosome binding per RBC (mean ± SEM, n≥3, ≥50 RBCs/condition). C) ddPCR shows increased RBC miR‐93‐5p after incubation with cancer cell‐derived exosomes vs controls (n = 3; mean ± SD; ^*^ *p* < 0.05; ^*^ ^*^ *p* < 0.01). D) Inhibition of exosome release from lung cancer cells with GW4869 reduces RBC loading of miR‐93‐5p compared with no GW4869 treatment; conditions included incubated with GW4869‐treated cancer cell‐derived exosomes, untreated cancer cell‐derived exosomes, and regular medium containing cancer cell‐derived exosomes. E) Inhibition of exosome uptake in RBCs with Dynasore diminishes miR‐93‐5p loading relative to controls; conditions included RBCs incubated with Dynasore‐treated cancer cell‐derived exosomes, untreated cancer cell‐derived exosomes, and regular medium containing cancer cell‐derived exosomes (n = 3). ^*^ *p* < 0.05; ^*^ ^*^ *p* < 0.01.

To investigate the cellular origin of miR‐93‐5p and whether it is produced by lung cancer cells and transferred to RBCs, exosomes were prepared from lung cancer cells, fluorescently labeled, and incubated with freshly isolated, platelet‐ and leukocyte‐depleted RBCs from healthy donors. RBCs exposed to cancer cell‐derived exosomes displayed distinct punctate signals (**Figure** **7**A), whereas RBCs incubated with exosomes from normal epithelial cells exhibited markedly fewer signals, and a negligible signal was detected with regular medium alone (Figure 7A,B). Consistently, incubation with cancer cell‐derived exosomes led to a significant increase in RBC miR‐93‐5p levels compared with exosomes from normal epithelial cells (Figure 7C). Importantly, this loading was markedly reduced by GW4869, an inhibitor of exosome release from NSCLC cells, and by Dynasore, an inhibitor of exosome uptake in RBCs (Figure 7D,E). These findings demonstrate that tumor‐derived exosomes are transferred to RBCs, thereby elevating RBC‐associated miR‐93‐5p. To extend these findings, we interrogated orthotopic lung cancer xenograft models. RBC‐exosomal miR‐93‐5p levels progressively increased in parallel with tumor growth and burden, as assessed by IVIS bioluminescence, demonstrating that RBC loading in mice could mirror tumor dynamics (Figure S11, Supporting Information). To validate these observations in human specimens, we analyzed paired tumor tissues, RBCs, and RBC‐derived exosomes from 226 lung cancer patients. Tumor miR‐93‐5p expression was strongly correlated with both RBC and RBC‐exosomal levels (Figure S12, Supporting Information), consistent with our earlier observation that miR‐93‐5p in RBCs and their exosomes, but not in plasma, was significantly associated with advanced disease stage (Tables S3 and S4, Supporting Information). Furthermore, in paired pre‐ and post‐surgery blood samples, which were available from 12 lung cancer patients, miR‐93‐5p levels decreased markedly in RBCs and their exosomes, but not in plasma, following tumor resection (Figure S13, Supporting Information). This suggests that tumor‐derived miR‐93‐5p is preferentially packaged into exosomes and transferred to RBCs. Taken together, these findings establish lung cancer cells as one of the primary sources of RBC‐associated miR‐93‐5p, transferred through exosomal communication.

### 2.10 RBC miR‐93‐5p Elevation Reflects Transfer from Tumor Exosomes Rather than De Novo Synthesis

To investigate how miR‐93‐5p becomes up‐regulated in the RBCs of lung cancer patients, we analyzed blood samples from 20 patients and 20 healthy controls. Hemolysis was assessed by A414 free hemoglobin, miR‐451/miR‐23a, and haptoglobin, with samples above prespecified thresholds flagged. In this sample set, 5 were hemolyzed and 35 were not (Figure S14A, Supporting Information). Exclusion of flagged samples did not change the patient‐control difference in RBC‐exosomal miR‐93‐5p, and adjusted models yielded similar results (Figure S14B,C, Supporting Information). Therefore, the upregulation of RBC‐exosomal miR‐93‐5p reflects true tumor biology rather than hemolysis artifacts.

We then fractionated exosomes from lung cancer patients and healthy controls into four groups: exosome‐all (total exosomes), exosome‐ΔEpCAM (depleted with anti‐EpCAM), exosome‐ΔEpCAM+Spike (reconstituted with captured EpCAM⁺ exosomes), and exosome‐IgG (isotype antibody control). All exosome fractions were normalized to equal particle numbers and subsequently incubated with RBCs from healthy donors. In lung cancer patient samples (Figure S15A,B, Supporting Information), depletion of EpCAM⁺ exosomes significantly reduced RBC miR‐93‐5p, while spike‐in restored the signal (*p* < 0.05). In contrast, exosomes from healthy controls (Figure S15C, Supporting Information) produced much lower baseline levels with minimal changes across conditions. Moreover, pri‐miR‐93‐1/‐2 and the miRNA‐processing enzymes DROSHA and DICER1 were undetectable in RBCs, whereas mature miR‐93‐5p increased after exposure to tumor‐derived exosomes (*p* < 0.05) (Figure S15D, Supporting Information). Altogether, these findings indicate that miR‐93‐5p is upregulated in RBCs through tumor‐derived exosomal transfer rather than de novo synthesis within RBCs.

### 2.11 RBC‐Exosome‐Mediated Tumor‐RBC‐Tumor Axis is Distinct From, Yet Complements, Conventional Autocrine and Paracrine Signaling

To determine whether the tumor‐RBC‐tumor relay represents a distinct signaling pathway or simply mirrors direct autocrine/paracrine signaling, we compared the effects of tumor‐derived exosomes and RBC‐derived exosomes from lung cancer patients on recipient NSCLC cells at equal particle doses. Both sources of exosomes were enriched in miR‐93‐5p. Recipient H460 cells were pre‐transfected with or without ASO‐miR‐93‐5p before exosome exposure. Recipient NSCLC cells exhibited a marked increase in exosome uptake following exposure to either tumor‐derived or RBC‐derived exosomes, compared with medium‐only controls (Figure S16A, Supporting Information). Furthermore, both sources of exosomes, enriched with miR‐93‐5p, reduced PTEN protein abundance (Figure S16B,C, Supporting Information), suppressed PTEN 3′‐UTR luciferase activity (Figure s16 D, Supporting Information), and enhanced NSCLC cell invasion and migration (Figure S16E,F, Supporting Information). In addition, ASO‐miR‐93‐5p treatment restored PTEN expression, reporter activity, and suppressed the tumorigenic phenotypes (Figure S16B–F, Supporting Information). These findings indicate that RBC‐derived exosomes transfer miR‐93‐5p with potency comparable to tumor exosomes, exhibiting functional equivalence and supporting a tumor‐RBC‐tumor axis that reinforces, rather than merely repeats, tumor‐derived signaling.

To confirm these observations, PTEN‐WT NSCLC cells were incubated with equal numbers of matched tumor‐derived or patient RBC‐derived exosomes in the presence of uptake modulators (heparin, anti‐integrin β1, anti‐integrin αv, and anti‐CD47 antibodies). Heparin reduced uptake of both sources (P < 0.05) (Figure S17A, Supporting Information), as did integrin β1/αv blockade, with proportional PTEN recovery (Figure S17B, Supporting Information) and PTEN‐3′UTR de‐repression (Figure S17C, Supporting Information; P < 0.05). Importantly, anti‐CD47, which blocks the CD47‐SIRPα inhibitory signaling axis on RBCs, selectively impaired RBC‐exosome uptake (P < 0.05) without affecting tumor‐exosome attachment and internalization. Isotype/Fc‐block treatments were functionally neutral, and cell viability was unchanged (all P>0.05). Consistently, CD47 blockade specifically rescued PTEN expression and restored PTEN‐3′ UTR luciferase activity suppressed by RBC‐derived exosomes. Both tumor‐derived and RBC‐derived exosomes rely on heparin‐ and integrin‐dependent entry, but only RBC exosomes additionally require CD47, a self‐recognition signal, thereby revealing a distinct uptake pathway. These findings further indicate that RBC‐derived exosomes do not merely duplicate the activity of tumor exosomes but rather complement it, providing a distinctive and critical mechanism for exosomal cargo uptake and functional delivery.

## 3 Discussion

Lung cancer remains the leading cause of cancer‐related mortality worldwide for both men and women.^[^ ^25^ ^]^ A deeper understanding of the molecular mechanisms driving lung carcinogenesis is crucial for developing effective diagnostic and targeted therapeutic strategies, ultimately leading to improved patient outcomes. RBCs constitute ≈45% of total blood volume and, despite lacking a nucleus, contain a diverse set of miRNAs.^[^ ^20^ ^]^ Recent work has expanded the biological scope of RBCs beyond their conventional roles.^[^ ^3^, ^4^, ^10^ ^]^ RBCs can affect tumor behavior, including promoting tumor cell invasiveness and altering immune responses.^[^ ^3^, ^4^, ^10^ ^]^ However, the molecular mechanisms underlying the contribution of RBCs to tumorigenesis remain to be fully elucidated.^[^ ^35^ ^]^ In this study, we demonstrate that RBCs promote lung cancer progression by transferring oncogenic miR‐93‐5p. Our results show that miR‐93‐5p is predominantly found in RBC‐derived exosomes, not in the Ago2 complex, indicating that exosomes serve as the primary vehicle for delivering this oncogenic miRNA from RBCs to lung cancer cells. Conversely, lung cancer cells release miR‐93‐5p‐rich exosomes that are transferred to RBCs, contributing to the accumulation of this oncogenic microRNA. Furthermore, inhibition of exosome release or blockade of exosome uptake significantly reduced miR‐93‐5p transfer and diminished its tumor‐promoting effects, supporting the centrality of exosome‐mediated communication in this process. These findings uncover a previously unrecognized bidirectional tumor‐RBC‐tumor communication axis, in which RBCs act as active carriers and enhancers of oncogenic signaling, thereby reinforcing malignant progression. Consequently, RBC‐associated miR‐93‐5p may serve as both a biomarker and a therapeutic target in lung cancer.

miR‐93‐5p is an oncomiR that can activate the PI3K/Akt pathway, being involved in cancer cell proliferation, invasion, and resistance to apoptosis.^[^ ^36^, ^37^ ^]^ It also acts as a target key regulator of the epithelial‐mesenchymal transition and Wnt signaling pathways.^[^ ^38^ ^]^ In line with these reports, our bioinformatics and luciferase reporter assays demonstrated that RBC‐derived miR‐93‐5p directly targets tumor suppressors such as PTEN, TP53INP1, THBS2, and CDKN1A. In this study, our bioinformatics analysis and luciferase reporter assays demonstrated that RBC‐derived miR‐93‐5p directly targets several tumor suppressor genes, including PTEN, upon delivery to lung cancer cells. Given PTEN's well‐established role in regulating cell proliferation, survival, and tumor suppression in lung cancer, we focused our investigations on elucidating the functional consequences of miR‐93‐5p‐mediated PTEN downregulation. Functional assays confirmed that miR‐93‐5p‐mediated suppression of PTEN significantly promoted lung cancer cell proliferation, migration, and invasion. These oncogenic effects were reversed by either PTEN overexpression or inhibition of miR‐93‐5p. Furthermore, in subcutaneous xenograft models, miR‐93‐5p promoted tumor growth, while treatment with ASO‐miR‐93‐5p led to significant tumor regression. These findings were further supported by results from an orthotopic lung cancer model, where cancer cells pretreated with miR‐93‐5p‐enriched exosomes formed significantly larger lung tumors. Notably, ASO‐miR‐93‐5p treatment in the orthotopic model not only reduced primary tumor volume but also significantly suppressed metastatic spread, as evidenced by decreased incidence of mediastinal lymph node and contralateral lung metastases, demonstrating its potential to inhibit tumor dissemination in vivo. Although miR‐451, another RBC‐derived miRNA, was also transferred to lung cancer cells via exosomes, it did not promote tumorigenic phenotypes, most likely due to its well‐established role as a housekeeping miRNA in RBCs, primarily involved in erythroid homeostasis.^[^ ^39^ ^]^ This contrast underscores the specificity of miR‐93‐5p in driving lung cancer progression.^[^ ^40^ ^]^ In addition to PTEN, our analysis identified other tumor suppressor genes, including TP53INP1, THBS2, and CDKN1A, as potential targets of miR‐93‐5p, suggesting broader regulatory roles in tumorigenic pathways. These findings highlight the multifaceted contribution of RBC‐derived exosomal miR‐93‐5p to lung cancer progression and reinforce its potential as a therapeutic target. RBC‐derived exosomes contain a diverse repertoire of miRNAs and other noncoding RNAs.^[^ ^21^ ^]^ In this study, we focused on miR‐93‐5p, as it was among the most significantly upregulated miRNAs in RBCs from lung cancer patients, based on our previous profiling analysis.^[^ ^26^ ^]^ Additional RBC‐derived miRNAs may also contribute to tumorigenesis, and their functional relevance warrants further investigation.

To investigate whether miR‐93‐5p in RBCs originates from lung cancer cells, we fluorescently labeled lung cancer cell‐derived exosomes and incubated them with RBCs from healthy donors. Labeled cancer cell‐derived exosomes were transferred to RBCs and elevated RBC miR‐93‐5p levels, with this loading markedly reduced by inhibitors of exosome release and endocytosis. Furthermore, in xenograft models, RBC‐exosomal miR‐93‐5p levels rose progressively with tumor growth and correlated strongly with tumor burden. In addition, analysis of a large lung cancer patient cohort revealed that tumor miR‐93‐5p expression correlated strongly with both RBC and the exosomal levels, which in turn were associated with advanced disease stage. Moreover, analysis of paired pre‐ and post‐surgery samples from 12 lung cancer patients showed that tumor resection markedly reduced RBC and exosomal miR‐93‐5p. Together, these findings provide strong evidence that miR‐93‐5p in RBCs originates from lung cancer cells through exosome‐mediated transfer.

To investigate how miR‐93‐5p becomes upregulated in RBCs of lung cancer patients, we first confirmed that this upregulation was not attributable to hemolysis, as hemoglobin concentration, miR‐451/23a ratio, and haptoglobin levels did not differ between lung cancer patients and healthy controls. Furthermore, depletion/reconstitution experiments demonstrated that tumor‐derived EpCAM⁺ exosomes were responsible for transferring miR‐93‐5p into RBCs, since removal of these exosomes reduced loading and spike‐in restored it. In addition, the absence of pri‐miR‐93 and the processing enzymes DROSHA and DICER1 in RBCs excluded de novo synthesis. The present findings, in combination with the above observations that RBC and exosomal miR‐93‐5p levels closely mirror tumor dynamics, rising with tumor growth, correlating with tumor burden and disease stage, and decreasing after tumor resection, suggest that the upregulation of miR‐93‐5p in RBCs of lung cancer patients results from the uptake of tumor‐derived exosomes. To further determine whether the tumor‐RBC‐tumor axis constitutes a distinct pathway or simply duplicates direct autocrine/paracrine signaling, we compared the effects of tumor‐derived exosomes and lung cancer patient‐derived RBC exosomes. Both sources efficiently delivered miR‐93‐5p to NSCLC cells, leading to PTEN suppression, reduced PTEN‐3′ UTR activity, and enhanced tumor aggressiveness, all of which were reversed by ASO‐miR‐93‐5p. Notably, uptake analyses showed that while both exosome types relied on heparin‐sensitive and integrin‐mediated entry, only RBC exosomes additionally required CD47, revealing a unique, selective uptake mechanism. Therefore, RBC exosomes do not simply duplicate tumor exosome signaling but instead complement it by presenting a distinct route of exosomal cargo uptake and functional delivery.

**Figure 8:**
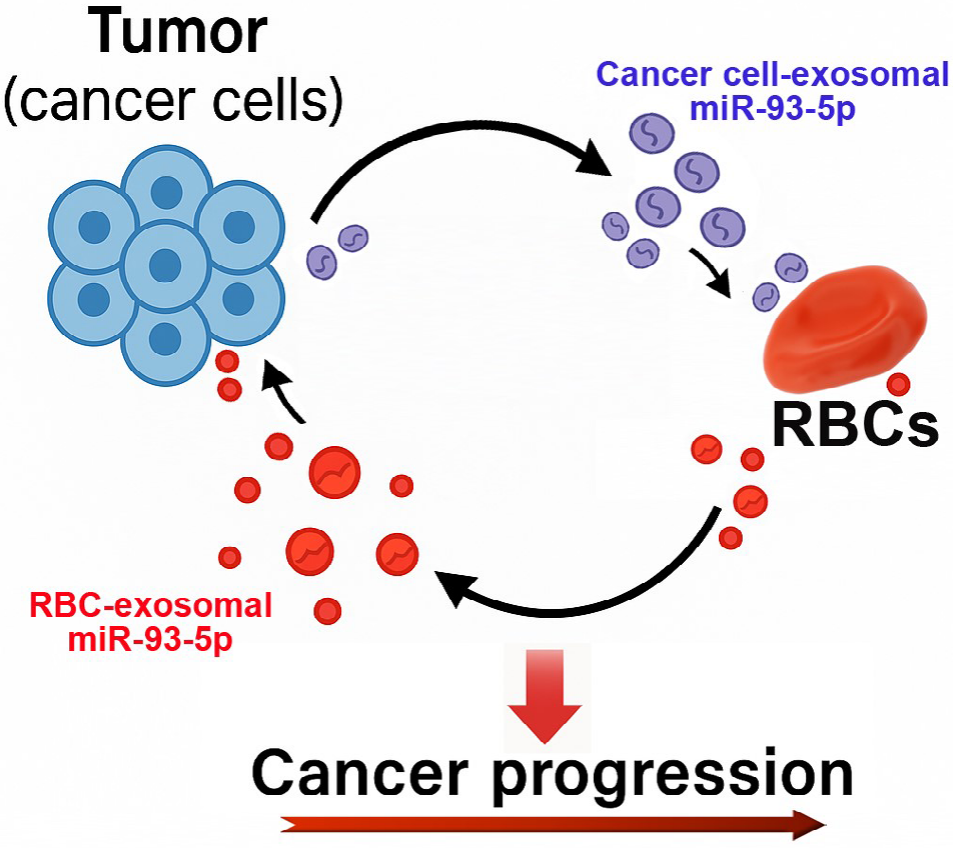
Schematic illustration of the tumor‐RBC‐tumor communication axis mediated by exosomal miR‐93‐5p in lung cancer. Tumor cells release exosomes enriched with miR‐93‐5p, which are taken up by RBCs. In turn, RBCs release exosomes containing miR‐93‐5p back to tumor cells, reinforcing oncogenic signaling and promoting tumor growth and metastasis. This bidirectional exchange highlights RBC‐associated miR‐93‐5p as a mechanistically distinct and functionally significant mediator of tumor progression in lung cancer. Because RBCs are abundant, long‐circulating, and capable of reaching tissues inaccessible to tumor cells and their exosomes, this RBC‐mediated pathway may play a particularly important role in promoting metastatic dissemination.

Overall, our findings demonstrate that the elevated levels of miR‐93‐5p in RBCs of lung cancer patients arise predominantly from the uptake of tumor‐derived exosomes. Functionally, RBC‐derived exosomes transfer miR‐93‐5p back to tumor cells, where they suppress PTEN expression and promote malignant phenotypes with potency comparable to that of tumor exosomes. RBC exosomes utilize a pathway distinct from that of tumor exosomes, establishing a mechanistically unique tumor‐RBC‐tumor communication axis (**Figure** **8**). The pathway may complement local signaling by extending the oncogenic communication systemically. Given that RBCs are the most abundant and long‐lived cells in circulation, this pathway positions them not as passive reservoirs reflecting tumor burden, but as active intermediaries that redistribute tumor‐derived oncomiRNAs through a route inaccessible to direct tumor–tumor or exosome‐cell communication. Because RBCs circulate systemically and can reach peripheral tissues and microenvironments that tumor cells and their exosomes are less likely to access, the RBC‐based pathway may play an especially important role in promoting tumor metastasis. Such RBC‐mediated transport extends the spatial and biological reach of oncogenic signaling, providing an alternative or complementary mechanism that facilitates metastatic dissemination. By broadening tumor‐host communication networks, RBCs and their associated exosomes of lung cancer patients may emerge as both valuable diagnostic and therapeutic targets. Nevertheless, alternative possibilities, such as RBCs acquiring miR‐93‐5p during erythropoiesis and retaining it throughout their lifespan, warrant further investigation.

## 4 Conclusion

Our study uncovers a previously unrecognized role of RBCs in lung cancer, demonstrating that they promote tumor progression through the exosomal transfer of oncogenic miR‐93‐5p. This process establishes a novel bidirectional tumor‐RBC‐tumor communication axis that reinforces malignant signaling and opens new avenues for diagnostic and therapeutic intervention.

## 5 Experimental Section

### Research Design

Peripheral blood was collected from histologically confirmed lung cancer patients and cancer‐free smokers. Plasma, RBCs, RBC‐derived exosomes, and surgically resected tumor tissues were sequentially isolated (Figure S1, Supporting Information) and analyzed by ddPCR to profile miRNAs, with a focus on miR‐93‐5p and its clinical relevance. In vitro, recipient non‐small cell lung cancer (NSCLC) cells were exposed to these exosomes to quantify miRNA uptake, evaluate PTEN repression, and assess downstream oncogenic phenotypes, including proliferation, invasion, and migration. Furthermore, RBCs from healthy donors were incubated with lung cancer cell‐derived exosomes to assess the transfer and loading of this oncomiRNA. In vivo, both subcutaneous xenografts and orthotopic lung cancer mouse models were employed to evaluate tumor growth and metastatic dissemination. Therapeutic inhibition of miR‐93‐5p was achieved using locked nucleic acid antisense oligonucleotides (ASO‐miR‐93‐5p). Finally, integrated molecular, histopathological, and survival analyses were conducted to determine whether RBC‐derived miR‐93‐5p drives tumor progression and whether its pharmacological silencing mitigates these effects.

### Patients and Specimens

The authors' study was approved by the Institutional Review Board of the University of Maryland, Baltimore (IRB HP‐00040666). NSCLC patients and cancer‐free smokers were recruited based on criteria complemented by demographic, radiological, and clinical data from their medical records.^[^ ^26^ ^]^ Blood samples were collected from all participants using standardized phlebotomy procedures in accordance with established clinical protocols.^[^ ^27^, ^28^, ^29^ ^]^ Briefly, peripheral venous blood was drawn into ethylenediaminetetraacetic acid (EDTA) ‐coated tubes (BD, Franklin Lakes, NJ) using a 21‐gauge needle to minimize shear stress and hemolysis. Samples were gently inverted 8‐10 times immediately after collection to mix with the anticoagulant and avoid clot formation. All specimens were processed within 2 h of collection to minimize RNA degradation and prevent cellular lysis. Subsequently, comprehensive molecular and biological analyses were conducted following the protocols described below. An overview of patient selection and sample processing workflow is provided in Figure S1 (Supporting Information). A total of 226 NSCLC patients and 239 cancer‐free smokers were enrolled (Table S1, Supporting Information). NSCLC diagnoses were confirmed by board‐certified pathologists using formalin‐fixed, paraffin‐embedded (FFPE) tumor tissue samples obtained through surgical resection or biopsy. Tumor classification and staging were based on the 8th edition of the American Joint Committee on Cancer (AJCC) TNM staging system.^[^ ^41^ ^]^ Histological subtypes—including adenocarcinoma (AC), squamous cell carcinoma (SCC), and large cell carcinoma (LC), were determined according to WHO classification criteria.^[^ ^33^, ^34^ ^]^ Staging was supplemented with imaging data (CT, PET‐CT, and/or MRI) and pathological reports at the time of diagnosis.^[^ ^42^, ^43^ ^]^ Cancer‐free smokers were defined as individuals with a ≥20 pack‐year smoking history and no clinical, radiographic, or pathological evidence of malignancy. Furthermore, frozen lung tumor tissues were collected, and corresponding FFPE sections were prepared to assess tumor cellularity, which was confirmed to be ≥70% by hematoxylin and eosin (H&E) staining. Complete clinical data, including diagnosis date, tumor characteristics, treatment regimens, and comorbidities, were obtained for all NSCLC patients. Patients were followed longitudinally through regular clinic visits, electronic health record updates, and linkage to the cancer registry. The following‐up duration ranged from 6 to 60 months. Overall survival (OS) was calculated from the date of pathological diagnosis to the date of death or last known follow‐up. Vital status and date of death were confirmed using hospital records, death certificates, and the Social Security Death Index.

### Cell Culture

Six human NSCLC cell lines, including A549 (adenocarcinoma^[^AC], harboring a missense mutation and partial loss of the PTEN gene; RRID:CVCL_0023), H226 (squamous cell carcinoma [SCC], PTEN wild‐type; RRID:CVCL_1546), H460 (large cell carcinoma [LC], PTEN wild‐type; RRID:CVCL_0459), H23 (AC, PTEN wild‐type; RRID:CVCL_1558), H520 (SCC, PTEN nonsense mutation; RRID:CVCL_1575), and H810 (LC, homozygous PTEN deletion; RRID:CVCL_1606), were obtained from the American Type Culture Collection (ATCC, Manassas, VA) (Table S2, Supporting Information). Two normal bronchial epithelial cell lines, NHBE (RRID:CVCL_7956) and BEAS‐2B (RRID:CVCL_0168), were also obtained from ATCC. All cell lines were cultured under ATCC‐recommended conditions to ensure consistency and integrity. All cell lines were authenticated via short tandem repeat (STR) profiling and routinely tested for mycoplasma contamination using the MycoAlert™ Mycoplasma Detection Kit (Lonza). All lines were confirmed to be contamination‐free before experiments.

### Droplet digital PCR (ddPCR)

ddPCR was employed to quantify miRNA and mRNA copy numbers in NSCLC cell lines, RBCs, RBC‐derived exosomes, plasma, lung tumor tissues from patients, and xenograft tissues.^[^ ^22^ ^]^ RNA was reverse‐transcribed using the TaqMan MicroRNA Reverse Transcription Kit (Thermo Fisher Scientific, Waltham, MA. Cat# 4 366 596) for miRNAs or High‐Capacity cDNA Reverse Transcription Kit (Thermo Fisher Scientific, Cat# 4 368 814) for mRNAs. ddPCR reactions were prepared in 20 µL volumes, containing 2× ddPCR Supermix for Probes (no dUTP; Bio‐Rad, Cat# 1 863 024), 250 nM TaqMan probe, 900 nM forward and reverse primers, and 2 µL of diluted cDNA template. Droplets were generated using the QX100 Droplet Generator (Bio‐Rad Laboratories, Hercules, CA), producing over 10000 droplets per well. PCR amplification was performed under the following conditions: 95 °C for 10 minutes (enzyme activation), followed by 40 cycles of 94 °C for 30 seconds (denaturation) and 60 °C for 1 minute (annealing/extension), with a final step at 98 °C for 10 minutes. After amplification, droplets were analyzed on the QX100 Droplet Reader using QuantaSoft Software (Bio‐Rad). Fluorescence amplitude thresholds were set manually based on no‐template controls to distinguish positive from negative droplets. The absolute concentration of target genes (copies/µL) was calculated using Poisson distribution statistics. Primer and probe sequences for miR‐93‐5p and PTEN were obtained from commercially available TaqMan Assays (Thermo Fisher Scientific). The ddPCR assay for miR‐93‐5p (Assay ID: 0 01090) targets the mature miRNA sequence 5′‐CAAAGUGCUGUUCGUGCAGGUAG‐3′, while the PTEN assay (Assay ID: Hs02621230_s1) is a pre‐designed gene expression assay with proprietary primer and probe sequences not publicly disclosed by the manufacturer. Assay IDs for pri‐miR‐93‐1 (Hs03303305_pri), pri‐miR‐93‐2 (Hs03303627_pri), DROSHA (Hs00203008_m1), and DICER1 (Hs00229023_m1) were used to quantify the precursors and biogenesis transcripts, respectively.

### Preparation of RBCs and RBC‐derived Exosomes

To ensure robust preparation of RBCs and RBC‐derived exosomes, we adhered to standardized protocols in accordance with Minimal Information for Studies of Extracellular Vesicles (MISEV) recommendations.^[^ ^26^, ^27^, ^28^, ^29^, ^30^, ^31^ ^]^ Within 2 h of blood collection, plasma was isolated by centrifugation at 1500 × g for 10 minutes at room temperature, followed by careful collection of the upper plasma layer. The plasma was clarified further by centrifugation at 2000 × g for 15 minutes at 4 °C to remove cellular debris and stored on ice for immediate use. To exclude hemolysis artifacts, plasma and RBC samples were assessed by A414 hemoglobin, miR‐451/23a ratio, and plasma haptoglobin; samples above thresholds were excluded. For selected experiments, RBC‐ and plasma‐derived exosomes were fractionated by size‐exclusion chromatography (SEC) (qEVoriginal columns, Izon Science, Medford, MA) and pooled based on nanoparticle tracking analysis (NTA) and protein absorbance at 280 nm. The buffy coat was manually removed to ensure a pure RBC pellet, which was washed twice with PBS (Sigma‐Aldrich, St. Louis, MO) to eliminate residual plasma and leukocytes. The washed RBC pellet was resuspended in hypotonic buffer (10 mM Tris‐HCl, pH 7.4) and incubated at room temperature for 30 minutes to lyse the cells. The lysate was centrifuged at 300 × g for 10 minutes to remove large debris, and the resulting supernatant was centrifuged at 16500 × g for 20 minutes at 4 °C to remove larger vesicles and contaminants. The final supernatant was subjected to ultracentrifugation at 100000 × g for 70 minutes at 4 °C using a Beckman Coulter ultracentrifuge (Optima XE‐90, Indianapolis, IN). The exosome pellet was washed with PBS, resuspended in 200 µL of PBS, and analyzed for size, concentration, and purity using nanoparticle tracking analysis and electron microscopy, following MISEV guidelines.^[^ ^31^ ^]^

### Preparation of Cancer cell‐derived Exosomes

For reliable preparation of cancer cell‐derived exosomes, we employed established protocols using differential clearing, ultracentrifugation, and characterization workflows as outlined above and in prior reports.^[^ ^26^, ^27^, ^28^, ^29^, ^30^, ^31^ ^]^ Briefly, cells were cultured to ∼70–80% confluence, washed twice with PBS, and switched to exosome‐depleted medium (RPMI with 10% exosome‐depleted FBS) for 24–48 h. Conditioned medium was collected on ice and processed identically to the RBC‐exosome pipeline: sequential centrifugation to remove cells/debris and large vesicles, 0.22‐µm filtration, pelleting of small exosomes by ultracentrifugation at 100000 × g, a PBS wash with re‐pelleting at 100000 × g, and final resuspension in PBS. Where higher purity was required, size‐exclusion SEC was applied. Particle size/concentration, morphology, and exosome identity were assessed as in the RBC‐exosome section. All steps were performed on ice or at 4 °C unless otherwise specified. For transfer vs de novo synthesis experiments, plasma‐derived EVs were incubated with anti‐EpCAM antibody‐coated magnetic beads (Miltenyi Biotec, Gaithersburg, MD) to deplete EpCAM⁺ tumor exosomes (exosome‐ΔEpCAM). EpCAM depletion/reconstitution was performed by eluting the captured fractions and spiking them back to the original particle number (exosome‐ΔEpCAM+Spike), while IgG‐coated beads (Thermo Fisher Scientific) served as isotype control.

### Electron Microscopy for the Analysis of Isolated Exosomes

The exosomal samples were fixed in 2.5% glutaraldehyde in 0.1 M cacodylate buffer (Sigma‐Aldrich). The samples then underwent a wash in cacodylate buffer (Sigma‐Aldrich) and were then dehydrated through a graded ethanol series (Sigma‐Aldrich). The exosome suspension was placed on microscopy grid (Ted Pella, Inc., Redding, CA) for 20 minutes. The sample was negatively stained with 2% uranyl acetate (Sigma‐Aldrich) for one minute. The grid was loaded into an Transmission Electron Microscopes (TEM) (Thermo Fisher Scientific) to capture images for size, shape, and aggregation of exosomes.

### Exosome Immunofluorescence, Release, Uptake, and Tracking in Cancer cell‐RBC Interactions

Purified exosomes were adsorbed onto glass slides, fixed with 4% paraformaldehyde (P6148, Sigma‐Aldrich) for 30 min at room temperature, washed with PBS, blocked in 3% BSA for 1 h, and incubated overnight at 4 °C with mouse anti‐CD63 antibody (1:100, clone TS63; ab8219, Abcam). After PBS washes, slides were incubated for 1 h at room temperature in the dark with goat anti‐mouse IgG (H+L)‐Alexa Fluor 488 (1:500, ab150113; Abcam), washed, mounted in antifade medium (F4680, Sigma‐Aldrich), and imaged by epifluorescence microscopy (Carl Zeiss Microscopy, White Plains, NY). Permeabilization (0.1% Triton X‐100, T8787, Sigma‐Aldrich) was omitted for surface epitopes and applied only when indicated, and negative controls included no primary antibody, isotype, and secondary‐only staining. To assess exosome release, cells were treated with GW4869 (10 µM; D1692, MilliporeSigma) for 24 h, and conditioned medium was collected and processed for exosome isolation by sequential centrifugation, filtration, and ultracentrifugation. For uptake inhibition, recipient cells were preincubated with Dynasore (80 µM; D7693, MilliporeSigma) for 30 min and maintained in its presence during co‐incubation, with exosome association confirmed by CD63 immunostaining (1:200; ab8219, Abcam). To determine which surface molecules mediate exosome uptake and thereby distinguish RBC‐exosome from tumor‐exosome entry, exosome uptake inhibition and receptor blockade were evaluated in H460 recipient cells. Cells were pretreated with heparin (20 µg/mL; Sigma), anti‐integrin β1 (clone P5D2; Abcam), anti‐integrin αv (clone L230; BioLegend, San Diego, CA), anti‐CD47 (clone B6H12; Abcam), or matched isotype IgG controls for 30 min prior to exosome exposure, with inhibitors maintained throughout incubation. Uptake was quantified by MemGlow labeling with trypan quenching, PTEN protein recovery was assessed by Western blot, and PTEN‐3′ UTR derepression (luciferase assay; Promega, Madison, WI) was measured using a luciferase reporter assay. To further evaluate cancer cell‐RBC interactions, purified exosomes were fluorescently labeled with directly conjugated anti‐CD63 antibody (2 µg per 1 × 10⁹ particles) for 30 min on ice, and unbound antibody was removed by SEC with pooled fractions containing exosomes. Labeled exosomes (1 × 10⁸ per 1 × 10⁶ RBCs) were then incubated with platelet‐ and leukocyte‐depleted RBCs for 2 h, washed, and visualized by epifluorescence microscopy, with RBCs stained using anti‐CD235a (clone HI264, Abcam) followed by Alexa Fluor 594‐conjugated secondary antibody (ab150080, Abcam) and nuclear dyes omitted. Surface‐bound fluorescence was minimized, with controls consisting of isotype‐labeled vesicles, antibody‐only SEC fractions, and unlabeled exosomes.

### Immunoprecipitation (IP) of Ago2

Samples were centrifuged at 10000 × g for 10 minutes to remove cellular debris as previously described.^[^ ^44^ ^]^ The supernatant was treated with protein extraction buffer containing RNase inhibitors (Sigma‐Aldrich, Cat# R8380) and incubated with protein A/G magnetic beads (Sigma‐Aldrich, Cat# M8823) for 1 h at 4 °C to pre‐clear nonspecific binding. An Ago2‐specific rabbit monoclonal antibody (Abcam, clone EPR10411, Catalog #ab32381) was added at a 1:100 dilution in extraction buffer and incubated overnight at 4 °C with gentle rotation. Fresh protein A/G magnetic beads were then added to capture the antibody‐antigen complexes by incubating for 2 h at 4 °C. After three washes with cold PBS or extraction buffer (Sigma‐Aldrich, Cat# P4417), the immunoprecipitated complexes were eluted using SDS sample buffer (Bio‐Rad, Cat# 1 610 747) and analyzed by Western blotting to confirm Ago2 protein enrichment, using GAPDH as a loading control.

### Cell Transfection

Synthetic constructs for miR‐93‐5p mimics, antisense oligoribonucleotide (ASO), and nonsense controls (NC) were designed and synthesized by Integrated DNA Technologies (IDT, Coralville, IA). The sequences were as follows: miR‐93‐5p mimic, Forward 5′‐CAAAGUGCUGUUCGUGCAGGUAG‐3′ and Reverse 5′‐CACCUGCACGAACAGCACUUUGU‐3′; NC mimic, Forward 5′‐UUCUCCGAACGUGUCACGUTT‐3′ and Reverse 5′‐ACGUGACACGUUCGGAGAATT‐3′; miR‐93‐5p ASO, 5′‐CACUUAUCAGGUUGUAUUAUAA‐3′; and NC ASO, 5′‐CAGUACUUUUGUGUAGUACAA‐3′.^[^ ^45^ ^]^ Constructs were transfected into lung cancer cells (1 × 10⁵ cells per well in 24‐well plates) using Lipofectamine RNAiMAX Transfection Reagent (Thermo Fisher Scientific, Cat# 13 778 150), following the manufacturer's protocol. Cells were incubated with the transfection complex for 6 h in serum‐free medium, after which the medium was replaced with complete growth medium and cultured for an additional 24–48 h at 37 °C with 5% CO_2_ prior to analysis. Transfection efficiency was confirmed by ddPCR, and all experiments were conducted in triplicate wells and repeated in at least three independent biological triplicate

### PTEN Overexpression via plasmid‐based Transfection

To achieve PTEN overexpression, a PTEN‐expressing plasmid (pcDNA3.1‐PTEN, Addgene Plasmid #20 741) under the control of the CMV promoter was used. The empty pcDNA3.1 vector served as a negative control. Cells were seeded at 0.5 × 10⁵ cells per well in 24‐well plates in complete DMEM medium (supplemented with 10% FBS, 100 U/mL penicillin, and 100 µg/mL streptomycin) and incubated overnight at 37 °C with 5% CO_2_. The next day, cells were transfected with 1 µg of plasmid DNA using Lipofectamine 2000 (Thermo Fisher Scientific, Cat# 11 668 019). DNA and Lipofectamine were diluted separately in Opti‐MEM (Gibco, Cat# 31 985 070), combined, incubated for 15 minutes at room temperature, and then added to serum‐free medium in each well. After 6 h, the medium was replaced with complete medium and cells were incubated for 24–48 h before downstream assays. Overexpression efficiency was confirmed by ddPCR and Western blotting.

### MTT Assay (3‐(4,5‐Dimethylthiazol‐2‐yl)‐2,5‐diphenyltetrazolium bromide)

Cells were seeded at a density of 5000 cells per well in 96‐well plates and cultured overnight at 37 °C with 5% CO_2_. After 24 h, 100 µL of medium containing 0.5 mg/mL MTT (Sigma‐Aldrich, Cat# M5655) was added to each well and incubated for 4 h. The medium was removed, and 100 µL of dimethyl sulfoxide (DMSO; Sigma‐Aldrich, Cat# D2650) was added to solubilize the formazan crystals. Absorbance at 570 nm was measured using a microplate reader (BioTek Synergy H1). Each condition was run in 4–6 replicate wells and normalized to vehicle‐treated controls. Experiments were repeated at least three times.

### Cell Invasion Assay

Cells (5 × 10⁴ cells in 200 µL serum‐free medium) were seeded into the upper chamber of a 24‐well Transwell insert (8 µm pore size; Corning, Cat# 3422) precoated with 50 µL of 1:4 diluted Matrigel (BD Biosciences, Cat# 354 234). The lower chamber contained 600 µL of complete medium with 10% FBS as a chemoattractant. After 24 h incubation at 37 °C, non‐invading cells were removed from the upper surface, and cells that invaded to the bottom surface were fixed with 100% methanol for 10 minutes, stained with 0.5% crystal violet (Sigma‐Aldrich, Cat# C0775) for 15 minutes, and counted in five random microscopic fields per membrane at 200× magnification.

### Cell Migration Assay

Cells (5 × 10⁴ cells in 200 µL serum‐free medium) were seeded into the upper chamber of uncoated 8 µm Transwell inserts (Corning, Cat# 3422). The lower chamber was filled with 600 µL of complete medium. After a 24 h incubation at 37 °C, migrated cells on the underside of the membrane were fixed with 4% paraformaldehyde (Sigma‐Aldrich, Cat# P6148) for 15 minutes, stained with 0.5% crystal violet, and counted in five randomly selected fields using an Olympus BX41 microscope. Assays were performed in triplicate.

### Dual Luciferase Reporter Assay

The PTEN 3′UTR, containing predicted miR‐93‐5p binding sites, was cloned into pmirGLO vectors (Promega, Madison, WI). Constructs were co‐transfected with miR‐93‐5p mimics, inhibitors, or controls into lung cancer cells (1 × 10⁵ cells per well). Dual‐luciferase activity was measured after 48 h using the Dual‐Luciferase Reporter Assay System (Thermo Fisher Scientific). Renilla luciferase was used for normalization.

### Western Blot Analysis

The PTEN 3′UTR containing predicted miR‐93‐5p binding sites was cloned into the pmirGLO Dual‐Luciferase miRNA Target Expression Vector (Promega, Cat# E1330). Constructs were co‐transfected into lung cancer cells (1 × 10⁵ cells per well in 24‐well plates) with miR‐93‐5p mimics, ASOs, or NC controls using Lipofectamine RNAiMAX. After 48 h of incubation, cells were lysed, and luciferase activity was measured using the Dual‐Luciferase Reporter Assay System (Thermo Fisher Scientific, Cat# 16 183) on a luminometer (Promega GloMax Discover). Firefly luciferase activity was normalized to Renilla luciferase as an internal control. All samples were run in triplicate wells, and experiments were repeated at least three times.

### Evaluation of the Oncogenic Role of RBC‐derived miR‐93‐5p Using a Subcutaneous Lung Cancer Xenograft Model

In accordance with an approved animal protocol (IACUC‐0516007) from the University of Maryland Baltimore, six‐week‐old female immunodeficient NOD. Athymic Swiss nu/nu/Ncr nu (nu/nu) mice were purchased from The Jackson Laboratory (Bar Harbor, ME). To evaluate the in vivo oncogenic potential of RBC‐exosomal miR‐93‐5p, mice were randomly assigned to either an experimental or control group (n = 5 per group). The experimental group received subcutaneous injections of 1 × 10⁵ NCI‐H460‐luc cells (TD2 Precision Oncology, Scottsdale, AZ) pre‐incubated with 50 ng/µL exosomes isolated from lung cancer patients with elevated miR‐93‐5p levels (confirmed by ddPCR). Control mice received H460‐luc cells incubated with 50 ng/µL exosomes derived from healthy donors. Tumor progression was monitored weekly for 4 weeks using the IVIS 200 Imaging System (Xenogen, Alameda, CA). Tumor size was measured by digital caliper in two perpendicular dimensions (Length and Width), and tumor volume was calculated using the formula: Volume = (Length × Width^2^) ÷ 2. Investigators assessing tumor size and histological outcomes were blinded to group identity to minimize bias. Endpoints were defined as tumor volume exceeding 1500 mm^3^, signs of distress or weight loss >20%, or 28 days post‐injection, whichever occurred first. Statistical comparisons of tumor volume between groups were performed using two‐way ANOVA. At study completion or upon reaching humane endpoints, mice were euthanized under deep pentobarbital anesthesia (Sigma‐Aldrich), and tumors were harvested for histopathological and molecular analyses. To test the therapeutic effect of miR‐93‐5p inhibition, 20 additional mice were injected with 1 × 10⁵ H460‐Luc cells and randomly assigned to receive weekly intraperitoneal injections of either ASO‐miR‐93‐5p or control ASO (50 mg/kg, AstraZeneca, Wilmington, DE). Tumor monitoring and volume assessment were conducted as described. Kaplan–Meier survival analysis with the log‐rank test was used to compare survival between groups.

### Functional Characterization and Therapeutic Targeting of RBC‐derived miR‐93‐5p in an Orthotopic Lung Cancer Model

To assess the role of RBC‐derived miR‐93‐5p in a more physiologically relevant tumor microenvironment, we utilized an orthotopic cancer model as previously described.^[^ ^46^ ^]^ NCI‐H460‐luc cells were pretreated with RBC‐derived exosomes from either healthy donors (low miR‐93‐5p) or lung cancer patients (high miR‐93‐5p) and suspended at 5 × 10⁵ cells in 25% growth factor‐reduced Matrigel. Mice were randomly allocated to groups (n = 5 per group), and cells were injected directly into the left lung parenchyma via transthoracic intrapulmonary injection using a microsyringe. Tumor progression was monitored weekly for 4 weeks by using the IVIS 200 Imaging System as described in the subcutaneous model. Investigators analyzing tumor burden and survival were blinded to treatment assignment. After 4 weeks or upon meeting predefined humane endpoints (respiratory distress, weight loss >20%, or moribund state), mice were euthanized, and lungs were collected. Paraffin‐embedded lung sections were prepared for downstream analysis. In a therapeutic cohort, mice bearing orthotopic tumors derived from high‐miR‐93‐5p‐pretreated cells received image‐guided intratumoral injections of ASO‐miR‐93‐5p or control ASO (50 mg/kg in PBS) via a microsyringe, administered two times per week for 3 weeks. To assess metastasis, thoracic tissues (contralateral lung lobes, mediastinal lymph nodes) were collected, fixed, and stained histologically; the presence of metastatic lesions was recorded and incidence rates compared. All procedures were conducted under institutional IACUC approval and adhered to NIH guidelines for animal care.

### Immunohistochemical Analysis

Tumor sections were incubated overnight at 4 °C with a primary rabbit monoclonal anti‐PTEN antibody (Abcam, Clone Y184, Catalog #ab32199, 1:100 dilution in antibody diluent buffer [Dako, Cat# S0809]). After washing with PBS, sections were incubated for 1 h at room temperature with an HRP‐conjugated goat anti‐rabbit IgG secondary antibody (Abcam, Catalog #ab6721, 1:500 dilution). Chromogen development was performed using 3,3′‐diaminobenzidine (DAB; Sigma‐Aldrich, Cat# D5637) for 5 minutes at room temperature, followed by counterstaining with Harris hematoxylin (Sigma‐Aldrich, Cat# H3136) for 1 minute. Slides were rinsed, dehydrated, cleared, and mounted with Cytoseal XYL mounting medium (Thermo Fisher Scientific, Cat# 8312‐4). Positively stained cells were quantified in five randomly selected high‐power fields (400×), and staining intensity was graded semi‐quantitatively on a scale from 0 (0–5% positive cells) to 3 (>50% positive cells) by two independent pathologists blinded to group identity.

### Enzyme‐linked Immunosorbent Assay (ELISA)

Tissue samples were homogenized in RIPA lysis buffer supplemented with protease inhibitors (Thermo Fisher Scientific, Cat# 89 900) and centrifuged at 14000 × g for 15 minutes at 4 °C to remove debris. Protein concentrations in supernatants were determined using the Pierce BCA Protein Assay Kit (Thermo Fisher Scientific, Cat# 23 225), following the manufacturer's instructions. Human PTEN protein levels were quantified using a sandwich ELISA kit (Abcam, Catalog #ab119522). Plates were pre‐coated with a capture monoclonal antibody specific to PTEN. Sample lysates (50 µL) were added in duplicate and incubated for 2 h at room temperature. After washing, a biotinylated mouse monoclonal anti‐PTEN detection antibody (Clone 6H2.1; Sigma‐Aldrich, Cat# 05‐240, 0.5 µg/mL) was added and incubated for 1 h. Wells were washed and incubated with HRP‐streptavidin (Abcam, Cat# ab7403, 1:5000) for 30 minutes, followed by development with TMB substrate (Thermo Fisher Scientific, Cat# 34 021) for 15 minutes. The reaction was stopped with 1N sulfuric acid (Thermo Fisher Scientific, Cat# 259 105), and absorbance was measured at 450 nm using a SpectraMax microplate reader (Molecular Devices). PTEN levels were normalized to total protein input and expressed as ng/mg of total protein.

### Bioinformatics Analysis for Gene Interaction

The interaction between lncRNAs and miRNAs was predicted using established bioinformatics tools. starBase 3.0 (http://starbase.sysu.edu.cn/) was utilized to identify lncRNA‐miRNA interactions based on validated and predicted data. miRDB (http://mirdb.org/) was employed to predict miRNA binding sites and functional annotations. Putative miRNA target genes were identified using TargetScan (http://www.targetscan.org) and miRDB, focusing on conserved binding sites and context scores. The results were further filtered and cross‐referenced with publicly available databases to prioritize biologically relevant interactions.

### Illumina MiSeq‐based Targeted Sequencing for PTEN Genomic Alterations

Frozen tumor tissue specimens were collected, and corresponding FFPE sections were prepared and used to confirm tumor cellularity, which was determined to be ≥70% based on hematoxylin and eosin (H&E) staining. Genomic DNA was extracted from tissues using the Qiagen QIAamp DNA Tissue Kit (Qiagen, Hilden, Germany) and assessed for concentration and quality using the NanoDrop spectrophotometer (Thermo Fisher Scientific) and the Qubit dsDNA HS Assay Kit (Thermo Fisher Scientific). DNA integrity was evaluated using the Agilent 2200 TapeStation (Agilent Technologies, Santa Clara, CA), and samples with a DNA integrity number (DIN) ≥ 3.0 were used for sequencing. Targeted sequencing of the PTEN gene, focusing on exons 5‐8, was performed using a custom panel designed for Illumina sequencing. Libraries were prepared using the Illumina DNA Prep Kit, following the manufacturer's protocol. Briefly, 10–50 ng of DNA was fragmented and tagged with sequencing adapters containing unique dual indices for sample multiplexing. After adapter ligation and enrichment, library quality and quantity were assessed using the BioAnalyzer 2100 (Agilent Technologies). Sequencing was conducted on the Illumina MiSeq platform using a 2 × 150 bp paired‐end read configuration, with a target sequencing depth of 500× or greater. Negative controls and blank samples were included to monitor potential contamination. Raw sequencing data were aligned to the GRCh38 human reference genome using BWA‐MEM, and somatic variants, including single nucleotide variants (SNVs), small insertions/deletions (indels), and copy number variations (CNVs), were identified using VarDict and annotated with ANNOVAR. Variants with a depth below 20× or an allele frequency below 5% were excluded. Coverage metrics were evaluated to confirm uniform sequencing across the targeted PTEN exons. Variants were classified according to the American College of Medical Genetics and Genomics (ACMG) guidelines and cross‐referenced with public databases such as dbSNP, ClinVar, and COSMIC. Statistical analysis was conducted to calculate the frequency of PTEN alterations across the cohort and within histological subtypes using the Chi‐square test or Fisher's exact test, with statistical significance set at *p* < 0.05.

### Statistical Analysis

All statistical analyses were conducted using GraphPad Prism version 9.0 (GraphPad Software, USA) and SPSS Statistics version 26.0 (IBM, USA), with statistical significance defined as *p* < 0.05. Data distribution was assessed by the Shapiro‐Wilk test for normality, and Levene's test was applied to evaluate homogeneity of variances; outliers were excluded only when attributable to technical error. Data are presented as mean ± standard deviation (SD) for normally distributed variables or as median with interquartile range (IQR) for non‐normally distributed variables, with error bars in figures reflecting the appropriate descriptive statistics. For comparisons between two groups, unpaired two‐tailed Student's t‐tests were used when assumptions were met, while the Mann–Whitney U test was applied for non‐parametric data. For multiple group comparisons, one‐way or two‐way ANOVA followed by Tukey's post hoc test was used for normally distributed data, and the Kruskal–Wallis test with Dunn's post hoc correction was used for non‐parametric data. Correlations between molecular markers and clinical parameters were analyzed by Pearson's correlation for continuous normally distributed variables with linear relationships or Spearman's rank correlation for non‐linear or ordinal data. Kaplan–Meier survival analysis with the log‐rank test was employed to compare survival outcomes in patient and animal studies. Unless otherwise specified, all assays included at least three independent biological replicates (n ≥ 3), and patient cohort analyses are reported with the exact sample sizes (n) in figure legends.

### Ethics Approval and Consent to Participate

The study was conducted under protocols approved by the Institutional Review Board of the University of Maryland Baltimore (IRB HP‐00040666), and all patients provided written informed

## Conflict of Interest

The authors declare no conflict of interest.

## Author Contributions

F.J. contributed to conceptualization, funding acquisition, and supervision, while J.L., P.D., V.H., and A.S. were involved in methodology, and the writing was carried out by J.L. and F.J.

## Supporting information



Supporting Information

## Data Availability

The data that support the findings of this study are available on request from the corresponding author. The data are not publicly available due to privacy or ethical restrictions.
