# Supplementary material for: Red Blood Cell‐Derived Exosomal miR‐93‐5p Promotes Lung Cancer Progression through PTEN Suppression
Source: Adv Sci (Weinh). 2025 Oct 29;13(3):e11940. doi: 10.1002/advs.202511940 (PMC12806425; doi:10.1002/advs.202511940)
Supplement: Supplementary file 1 — Supporting Information [file ADVS-13-e11940-s001.pdf]

Supplementary Files

**Table S1.** Characteristics of 226 NSCLC patients and 239 cancer-free smokers		
Characteristics	NSCLC cases (n = 226)	cancer-free smokers (Controls) (n = 239)
Age (Mean, SD)	68.37 (12.17)	66.23 (11.53)
Sex - Female	77 (34.1%)	86 (36.0%)
Sex - Male	149 (65.9%)	153 (64.0%)
Race - African Americans (AAs)	83 (36.7%)	86 (36.0%)
Race - White Americans (WAs)	143 (63.3%)	153 (64.0%)
Smoking pack-years (median)	36	28
Stage I	66 (29.2%)	
Stage II	126 (55.8%)	
Stage III-IV	34 (15.0%)	
Histological type - Adenocarcinoma (AC)	113 (50.0%)	
Histological type - Squamous cell carcinoma (SCC)	90 (39.8%)	
Histological type - large cell carcinoma (LC)	23 (10.2%)	
Abbreviations: NSCLC, non-small cell lung cancer. SD, standard deviation.		

**Table S2.** Lung Cancer and Normal Bronchial Epithelial Cell Lines			
	Cell Line	Histology	PTEN Status (Genomic Alteration)
1	A549	AC	Wild-type (parental)
2	NCI-H226	SCC	Wild-type
3	NCI-H460	LC	PTEN wild-type
4	NCI-H23	AC	PTEN mutated
5	NCI-H520	SCC	PTEN mutated (nonsense mutation)
6	NCI-H810	LC	PTEN deleted (homozygous deletion)
7	NHBE	Normal	PTEN wild-type
8	BEAS-2B	Normal	PTEN wild-type

**Table S3:** Correlation of miR-93-5p in RBCs and Exosomes with Clinical and Demographic Characteristics of NSCLC Patients		
Characteristic	Spearman's correlation (ρ)	p-value
miR-93-5p in RBCs vs. Tumor Stage (I)	0.423	**0.031**
miR-93-5p in RBCs vs. Tumor Stage (II)	0.582	**0.017**
miR-93-5p in RBCs vs. Tumor Stage (III-IV)	0.735	**0.002**
miR-93-5p in Exosomes vs. Tumor Stage (I)	0.396	**0.045**
miR-93-5p in Exosomes vs. Tumor Stage (II)	0.541	**0.022**
miR-93-5p in Exosomes vs. Tumor Stage (III-IV)	0.694	**0.005**
miR-93-5p in RBCs vs. Age	0.157	0.421
miR-93-5p in RBCs vs. Smoking Pack-Years	0.123	**0.032**
miR-93-5p in RBCs vs. Histological Type	0.183	0.328
miR-93-5p in Exosomes vs. Age	0.179	0.375
miR-93-5p in Exosomes vs. Smoking Pack-Years	0.118	**0.026**
miR-93-5p in Exosomes vs. Histological Type	0.145	0.489
miR-93-5p in RBCs vs. Sex (Male)	0.073	0.721
miR-93-5p in RBCs vs. Sex (Female)	0.055	0.812
miR-93-5p in RBCs vs. Race (AAs)	0.087	0.665
miR-93-5p in RBCs vs. Race (WAs)	0.064	0.754
miR-93-5p in Exosomes vs. Sex (Male)	0.092	0.632
miR-93-5p in Exosomes vs. Sex (Female)	0.045	0.878
miR-93-5p in Exosomes vs. Race (AAs)	0.072	0.693
miR-93-5p in Exosomes vs. Race (WAs)	0.059	0.804
Spearman's rank correlation was used for analyzing associations between miR-93-5p levels in RBCs and exosomes with NSCLC characteristics.		

**Table S4:** Associations between RBC-miR-93-5p in RBCs and exosomes and clinical and demographic data, analyzed using Pearson's correlation coefficients.								
	RBC-miR-93-5p	Exosomal miR-93-5p	Age	Gender	Race	Pack-smoking years	Types of cancer	Stage
RBC-miR-93-5p	1	**0.023**	0.242	0.071	0.068	**0.019**	0.262	**0.048**
Exosomal miR-93-5p		1	0.155	0.239	0.054	**0.025**	-0.159	**0.029**
Age			1	-0.304	0.127	0.115	0.293	0.094
Gender				1	1	0.327	0.218	0.125
Race						0.475	0.321	0.129
Pack-smoking years						1	0.143	0.203
Types of cancer							1	0.137
Stage								1

**Table S5:** Multivariable Cox regression analysis of miR-93-5p and miR-451 in RBCs, exosomes, and plasma adjusted for tumor stage and smoking history.			
Variable	Hazard Ratio (HR)	95% CI	p-value
RBC miR-93-5p	1.85	1.25-2.74	**0.002**
Exosomal miR-93-5p	1.72	1.10-2.69	**0.015**
Plasma miR-93-5p	1.4	0.90-2.18	0.11
RBC miR-451	0.95	0.62-1.45	0.81
Exosomal miR-451	1.02	0.70-1.49	0.91
Plasma miR-451	1.1	0.75-1.62	0.44
Tumor stage	2.1	1.40-3.15	**0.001**
Smoking (pack-years)	1.35	1.05-1.72	**0.022**

In multivariable Cox regression analysis adjusted for tumor stage and smoking history, RBC and exosomal miR-93-5p remained significant predictors of overall survival, while plasma miR-93-5p showed a nonsignificant trend (Table S5). Tumor stage was a strong independent predictor, and smoking history contributed as a moderate but significant covariate. miR-451 levels in RBCs, exosomes, and plasma were not associated with outcome.

**Table S6:** Correlation of miR-451 in RBCs and Exosomes with Clinical and Demographic Characteristics of NSCLC Patients		
Characteristic	Spearman's correlation (ρ)	p-value
miR-451 in RBCs vs. Tumor Stage (I)	0.083	0.652
miR-451 in RBCs vs. Tumor Stage (II)	0.125	0.489
miR-451 in RBCs vs. Tumor Stage (III-IV)	0.152	0.374
miR-451 in Exosomes vs. Tumor Stage (I)	0.097	0.621
miR-451 in Exosomes vs. Tumor Stage (II)	0.115	0.502
miR-451 in Exosomes vs. Tumor Stage (III-IV)	0.149	0.415
miR-451 in RBCs vs. Age	0.067	0.732
miR-451 in RBCs vs. Smoking Pack-Years	0.075	0.685
miR-451 in RBCs vs. Histological Type	0.052	0.791
miR-451 in Exosomes vs. Age	0.094	0.643
miR-451 in Exosomes vs. Smoking Pack-Years	0.086	0.699
miR-451 in Exosomes vs. Histological Type	0.063	0.826
miR-451 in RBCs vs. Sex (Male)	0.027	0.934
miR-451 in RBCs vs. Sex (Female)	0.036	0.889
miR-451 in RBCs vs. Race (African Americans)	0.058	0.795
miR-451 in RBCs vs. Race (White Americans)	0.063	0.762
miR-451 in Exosomes vs. Sex (Male)	0.075	0.905
miR-451 in Exosomes vs. Sex (Female)	0.059	0.873
miR-451 in Exosomes vs. Race (African Americans)	0.061	0.748
miR-451 in Exosomes vs. Race (White Americans)	0.053	0.812

**Table S7:** Clinical and Demographic Characteristics of 26 Lung Cancer Patients with Elevated miR-93-5p Levels and 26 Healthy Controls		
Characteristics	Lung Cancer Patients (n = 26)	Healthy Controls (n = 26)
Age (Mean ± SD)	68.4 ± 12.2	66.2 ± 11.5
Sex		
Female	9 (34.6%)	10 (38.5%)
Male	17 (65.4%)	16 (61.5%)
Race		
AAs	9 (34.6%)	10 (38.5%)
WAs	17 (65.4%)	16 (61.5%)
Smoking pack-years (Median)	32	27
Stage		
Stage I	8 (30.8%)	
Stage II	14 (53.8%)	
Stage III-IV	4	
Histological Type		
AC	13 (50%)	
SCC	10 (38%)	
LC	3 (12%)	

**Table S8.** Frequency of PTEN Genomic Alterations by NSCLC Stage				
	Missense Mutations	Truncating Mutations	Frameshift/Nonsense Mutations	Splice Site Mutations
Stage I (%)	0.81	0.65	0.54	0.27
Stage II (%)	1.08	0.81	0.65	0.43
Stage III-IV (%)	2.05	1.62	1.35	0.86

**Table S9**. PTEN Mutation Percentages by Histology				
	Missense Mutations	Truncating Mutations	Frameshift/Nonsense Mutations	Splice Site Mutations
Adenocarcinoma (AC)	1.37	1.23	0.82	0.69
Squamous Cell Carcinoma (SCC)	1.71	1.58	1.23	0.82
Large Cell Carcinoma (LC)	1.03	0.34	0.21	0.07

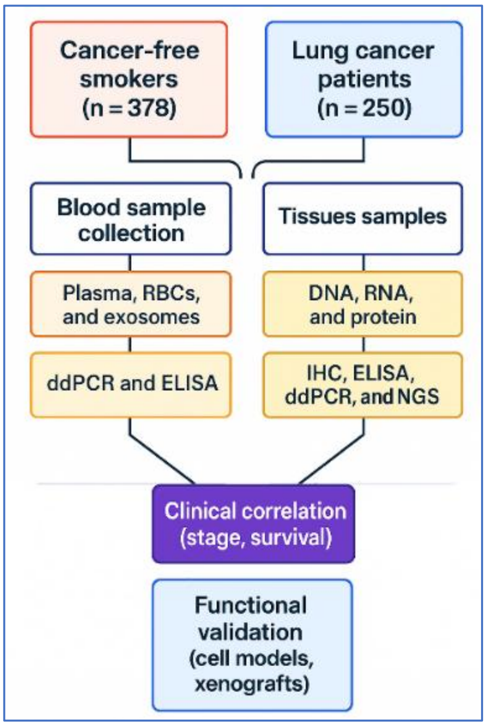

Figure S1. Flowchart summarizing the study design, including the selection of cancer-free smokers (n = 378) and lung cancer patients (n = 250).
Blood samples from both groups were processed to isolate plasma, RBCs, and exosomes, while tissue samples from lung cancer patients were analyzed for DNA, RNA, and protein content. Molecular analyses included ddPCR, ELISA, IHC, and NGS. Clinical correlations (e.g., tumor stage and patient survival) and functional validations using in vitro cell models and in vivo xenografts were conducted to investigate the biological and clinical relevance of the molecular findings.

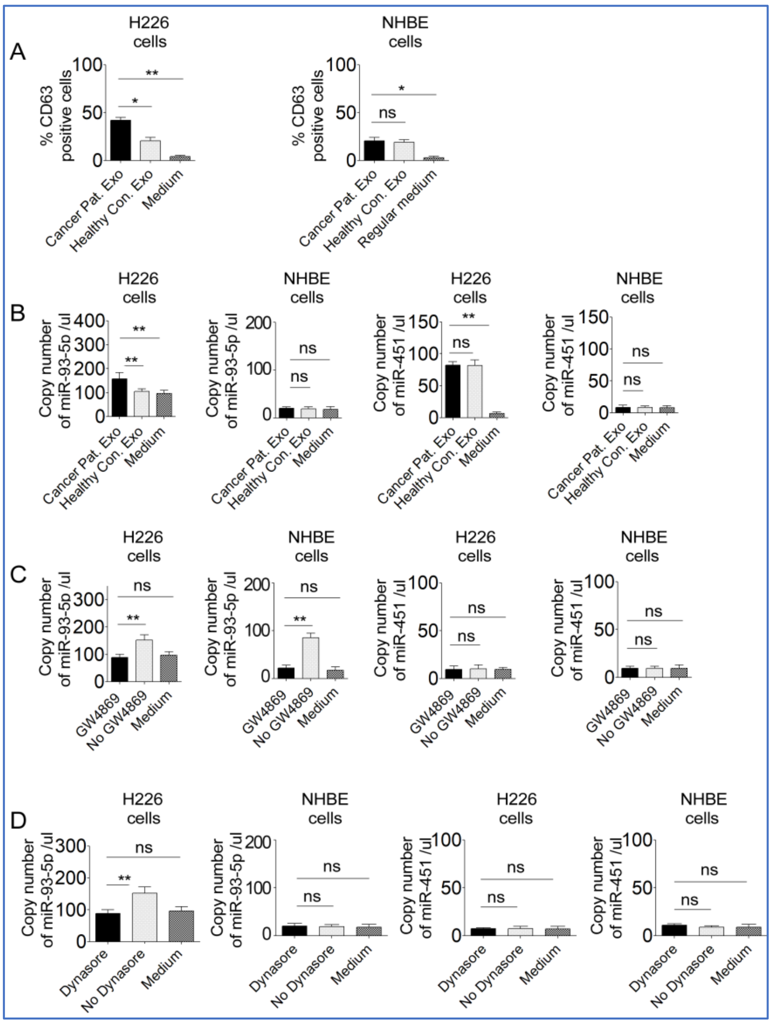

Figure S2. RBC-derived exosomes mediate miRNA transfer to NSCLC cells.
(A) Immunofluorescence staining reveals increased CD63-positive puncta in H226 lung cancer and NHBE normal cells treated with exosomes from lung cancer patients compared to those treated with exosomes from healthy controls. (B) ddPCR quantification shows elevated intracellular levels of miR-93-5p and miR-451 in NSCLC and NHBE cells exposed to cancer-derived exosomes, whereas exosomes from healthy donors induced miR-451 only. (C) RBCs pre-treated with GW4869 failed to release CD63-positive exosomes or transfer miRNAs to NSCLC and NHBE cells. (D) Dynasore treatment inhibited exosome uptake and miRNA delivery, supporting internalization-dependent transfer. Statistical analyses were performed using Student's t-test. Data are presented as mean ± SD. *, $p < 0.01$; **, $p < 0.001$. Three NSCLC cell lines were tested, all showing consistent results.

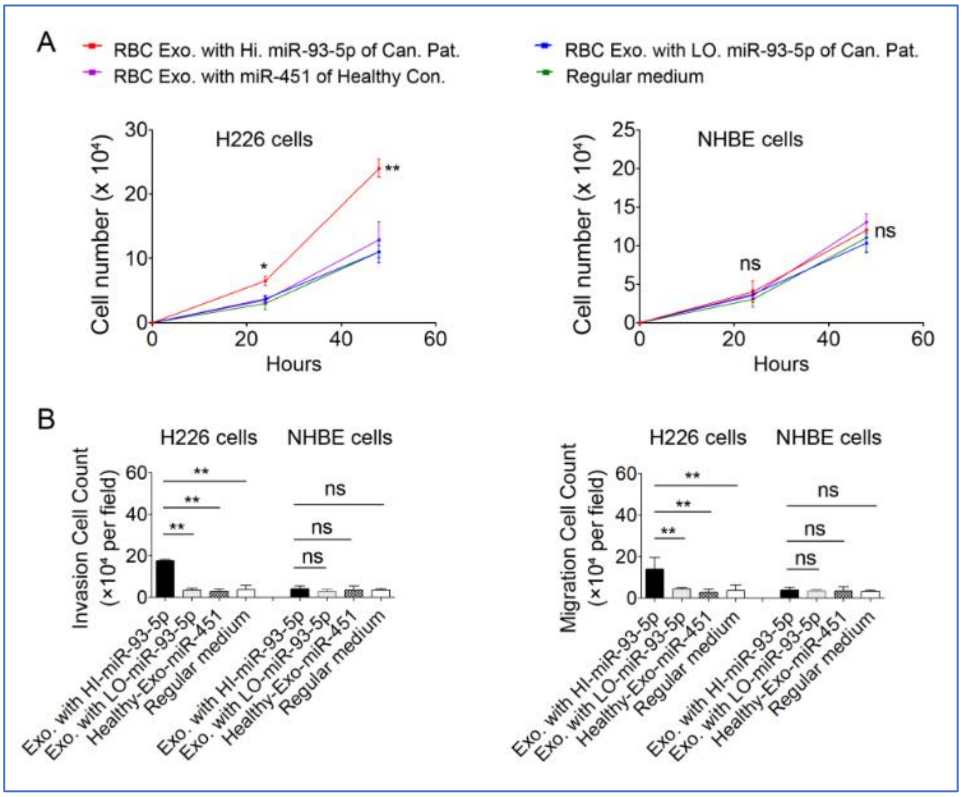

Figure S3. RBC-derived exosomal miR-93-5p promotes malignant phenotypes in NSCLC cells but not in normal bronchial epithelial cells.
(A) Cell proliferation of H226 (NSCLC) and NHBE cells after treatment with RBC-derived exosomes from lung cancer patients with high or low miR-93-5p levels, or from healthy donors. H226 cells treated with high-miR-93-5p exosomes showed significantly increased proliferation. (B) Quantification of invasion (left) and migration (right) of H226 and NHBE cells under the same treatment conditions. Only H226 cells exposed to high-miR-93-5p exosomes exhibited enhanced invasion and migration. Exosomes enriched in miR-451 had no effect, confirming the specificity of miR-93-5p. Data are shown as mean ± SD; *$p < 0.05$, **$p < 0.01$; ns, not significant.

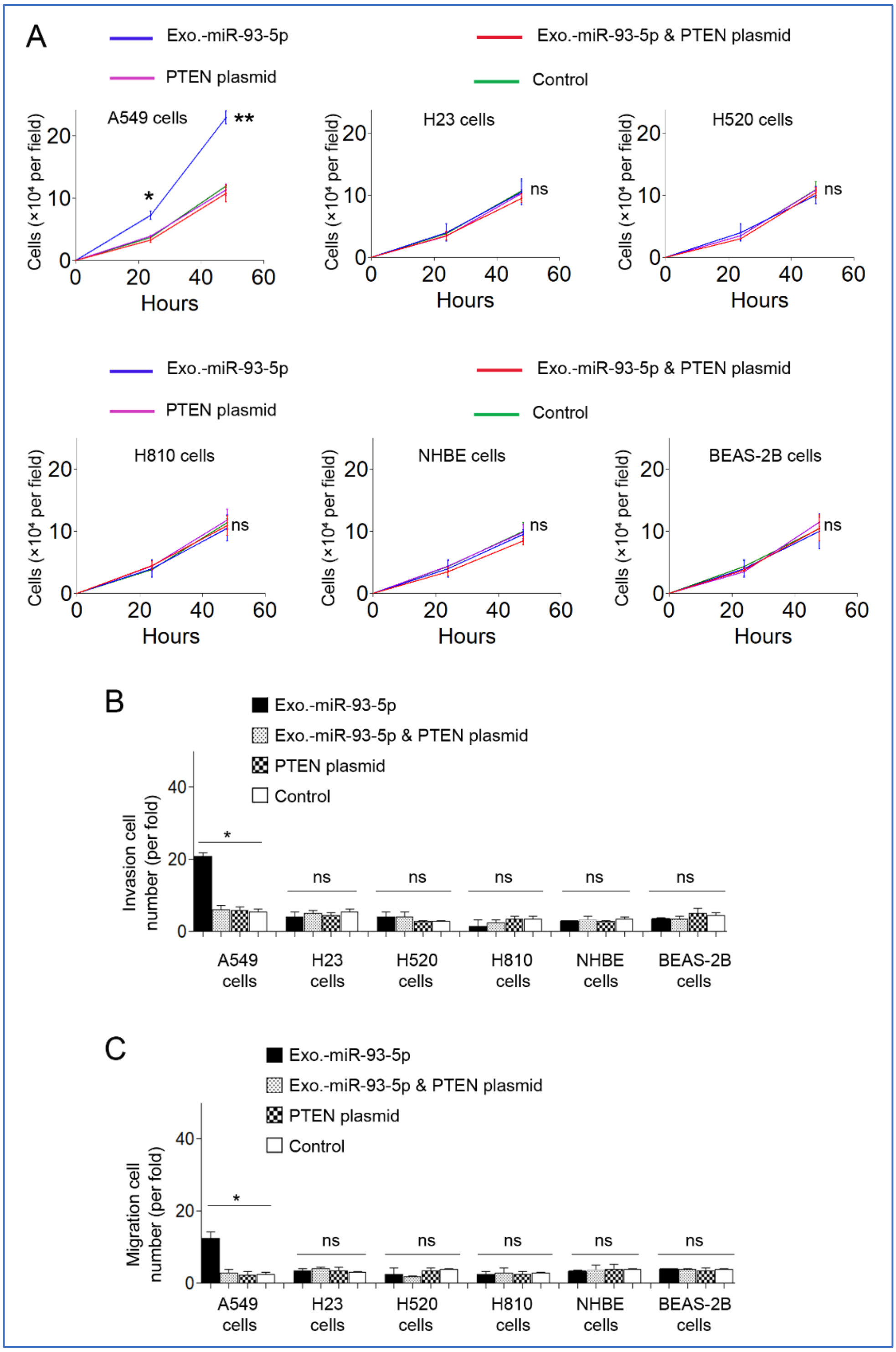

Figure S4. NSCLC and normal bronchial epithelial cells with PTEN mutations or deletions exhibit minimal phenotypic response to miR-93-5p–enriched RBC-derived exosomes.
(A) Cell proliferation assays were performed in NSCLC cell lines with PTEN mutations or deletions (H23, H520, H810), as well as in normal bronchial epithelial cells (NHBE and BEAS-2B), following treatment with exosomes enriched in miR-93-5p, with or without PTEN plasmid co-transfection. A549 cells (PTEN wild-type) were included as a positive control and showed significantly increased proliferation in response to miR-93-5p. (B) Cell invasion assays and (C) Migration assays revealed minimal changes in the PTEN-deficient NSCLC lines and normal epithelial cells compared to controls, whereas A549 cells exhibited a marked increase in invasive and migratory behavior upon miR-93-5p exosome treatment. Data represent mean ± SD from three independent experiments; statistical comparisons were conducted using unpaired two-tailed Student's t-tests. *, $p < 0.05$; **, $p < 0.01$; ns, not significant.

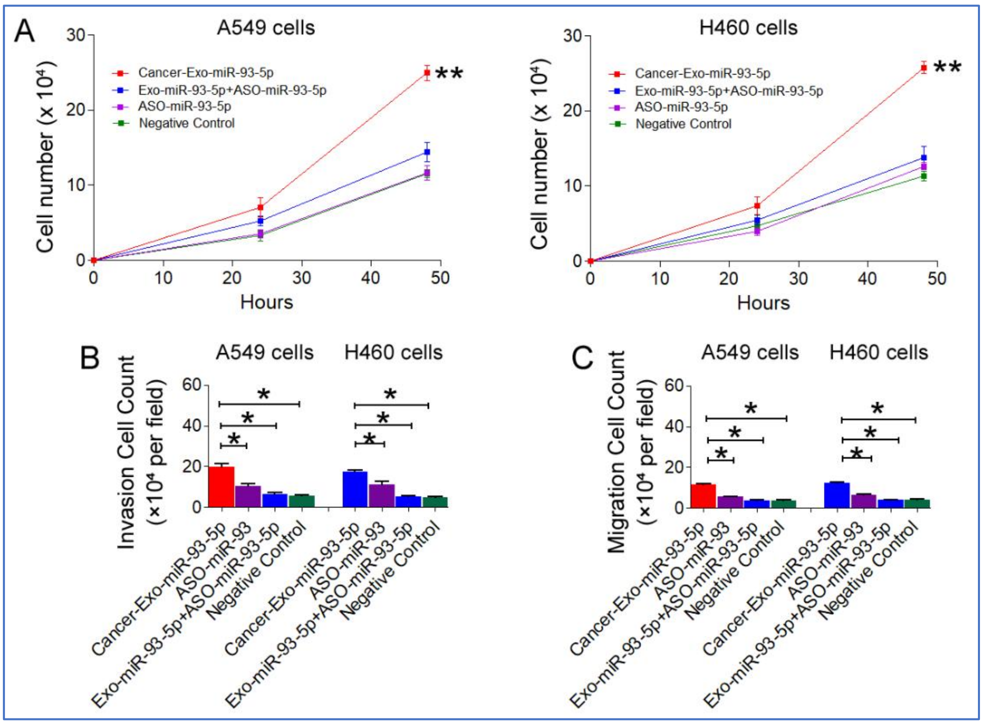

Figure S5. Effects of miR-93-5p-rich RBC-exosomes and ASO-miR-93-5p on tumorigenicity of lung cancer cells.

A) Cells treated with cancer-derived exosomes enriched in miR-93-5p (Cancer-Exo-miR-93-5p) have increased proliferation over time, compared to the negative control. The ASO-miR-93-5p treatment reduces cell numbers compared to cells treated with miR-93-5p-rich exosomes, approaching the levels of regular medium. B) Invasion assay results for cancers cells treated with RBC-exosomes rich in miR-93-5p from lung cancer patients, ASO-miR-93-5p, and control conditions, represented as the fold change relative to the negative control. In both cell lines, RBC-exosomes rich in miR-93-5p from lung cancer patients enhance invasion capabilities, which are significantly decreased by ASO-miR-93-5p treatment. C) Migration assay results for cancer cells under the same treatment conditions as in (B), showing a similar trend where miR-93-5p increases cell migration, and this effect is mitigated by ASO-miR-93-5p. Error bars indicate standard deviation. *, $p<0.05$, **, $p<0.01$.

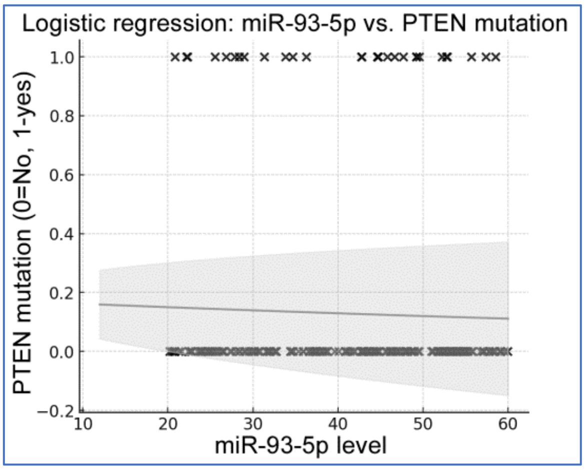

Figure S6: Scatter Plot Illustrating the Association Between miR-93-5p Expression and PTEN Status.

Each black dot represents an individual case, plotting miR-93-5p expression against binary PTEN status (wild-type vs. altered). The solid dark line represents the logistic regression trendline, estimating the probability of PTEN alterations as a function of miR-93-5p levels. The surrounding shaded region delineates the 95% confidence interval for the regression line. The analysis indicates a statistically non-significant correlation between miR-93-5p levels and PTEN mutations.

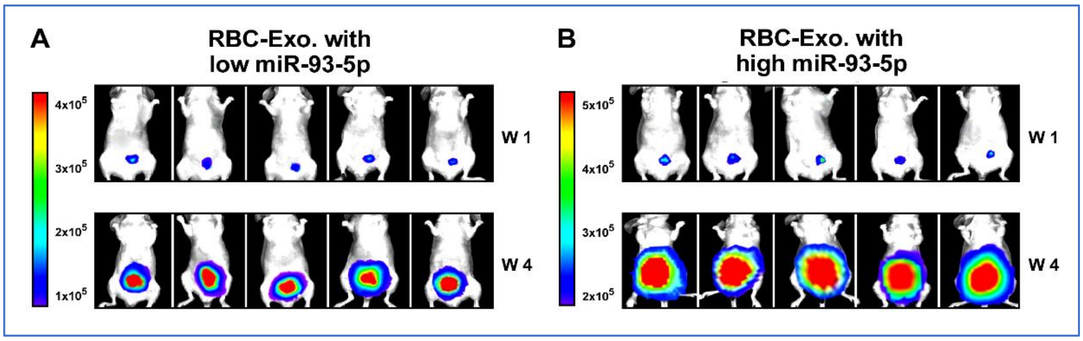

Figure S7. Bioluminescence imaging was conducted at Week 1 (W1) and Week 4 (W4) to monitor tumor progression.
Mice injected with H460-Luc cells pre-treated with RBC-derived exosomes enriched in miR-93-5p displayed significantly higher tumor burden at W4, as indicated by increased photon flux intensity, compared to mice treated with exosomes containing low levels of miR-93-5p. The heatmap scale reflects photon emission, with higher intensity corresponding to greater tumor load. (A) Representative images from mice treated with low miR-93-5p RBC exosomes. (B) Representative images from mice treated with high miR-93-5p RBC exosomes.

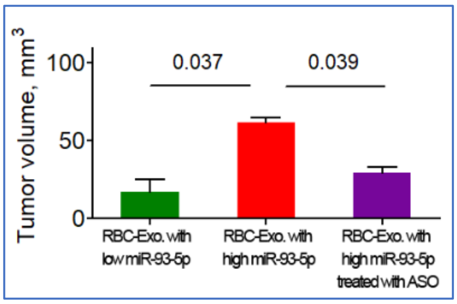

Figure S8. Tumor volume analysis of orthotopic lung tumors formed from H460 NSCLC cells. Mice injected with cells pretreated with RBC-derived exosomes enriched in miR-93-5p developed significantly larger tumors compared to the low-miR-93-5p group ($p = 0.037$). Treatment with ASO-miR-93-5p reduced tumor volumes in the high-miR-93-5p group ($p = 0.039$). Data are presented as mean ± SD.

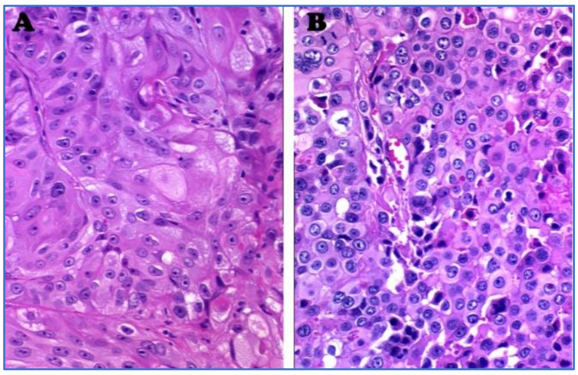

Figure S9. Representative H&E-stained sections showing metastatic lesions in the orthotopic lung cancer model implanted with H460 NSCLC cells (large cell carcinoma subtype).
(A) Metastatic tumors in the contralateral lung lobes display solid sheets and nests of tumor cells with large polygonal morphology, abundant cytoplasm, vesicular nuclei, and prominent nucleoli. The absence of glandular, squamous, or neuroendocrine differentiation is consistent with LC. Magnification: ×200. (B) Metastatic tumors in mediastinal lymph nodes exhibit poorly differentiated tumor cells with high nuclear-to-cytoplasmic ratios, open chromatin, and conspicuous nucleoli. Lack of gland formation, keratinization, or neuroendocrine features supports the diagnosis of LC. Magnification: ×200.

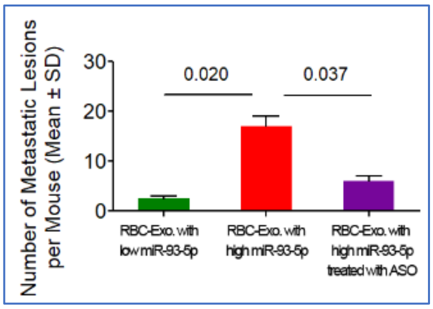

Figure S10. Quantification of metastatic lesions in the orthotopic lung cancer model. Mice injected with H460 NSCLC cells pretreated with RBC-derived exosomes enriched in miR-93-5p developed significantly more metastatic lesions compared to the low-miR-93-5p group ($p = 0.020$). ASO-miR-93-5p therapy reduced the number of metastatic lesions per mouse in the high-miR-93-5p group ($p = 0.037$). Data are presented as mean ± SD.

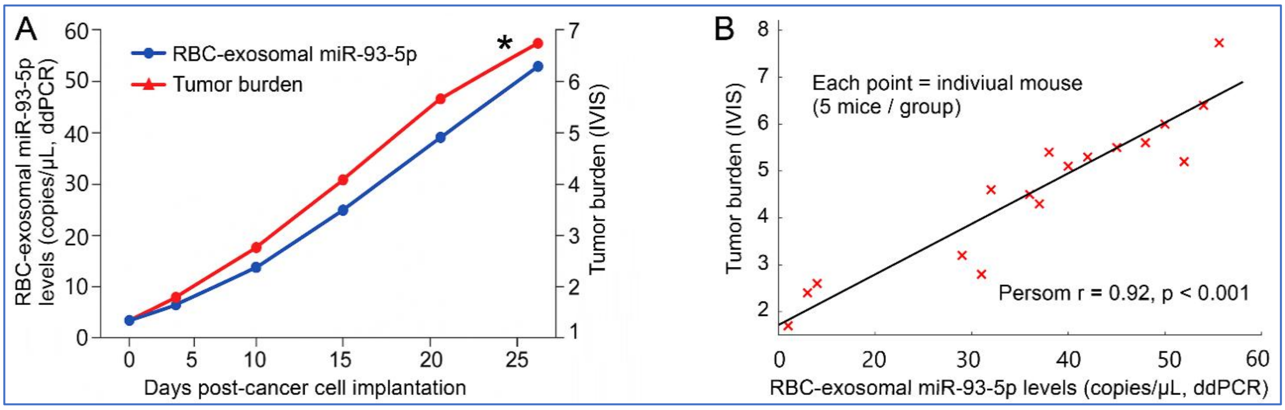

Figure S11. Dynamics of RBC-exosomal miR-93-5p and its correlation with tumor burden in orthotopic lung cancer xenograft models. (A) Time-course analysis of RBC-exosomal miR-93-5p levels (blue line, left Y-axis) and tumor burden, assessed by IVIS bioluminescence imaging (red line, right Y-axis), in mice following orthotopic implantation of H460-Luc NSCLC cells. Measurements were obtained over 25 days post-implantation. Both RBC-exosomal miR-93-5p and tumor burden increased significantly over time in parallel, as determined by a mixed-effects model (*, $p < 0.001$). (B) Correlation analysis between RBC-exosomal miR-93-5p levels and tumor burden (IVIS signal). Each red cross represents an individual mouse at a given measurement point ($n = 5$ mice per group). A strong positive correlation was observed (Pearson $r = 0.92$, $p < 0.001$). Data shown are representative time points collected within 25 days post-implantation.

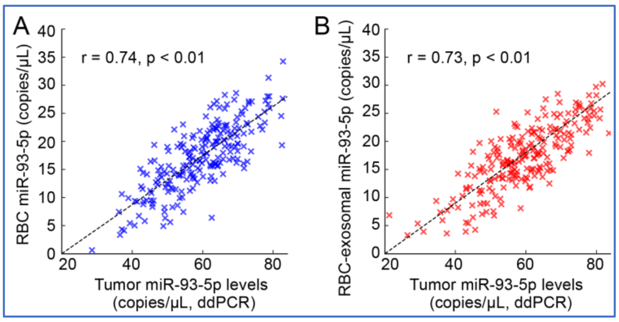

Figure S12. Correlation of tumor miR-93-5p expression with RBC and RBC-exosomal levels in 226 lung cancer patients (A) Scatterplot of tumor versus RBC miR-93-5p levels (copies/μL, ddPCR). Each point represents an individual patient, with a strong positive correlation (Pearson $r = 0.74$, $p = 0.0002$). (B) Scatterplot of tumor versus RBC-exosomal miR-93-5p levels. A similar strong correlation was observed (Pearson $r = 0.73$, $p = 0.0001$). All statistical analyses were performed using Pearson correlation with two-tailed significance testing.

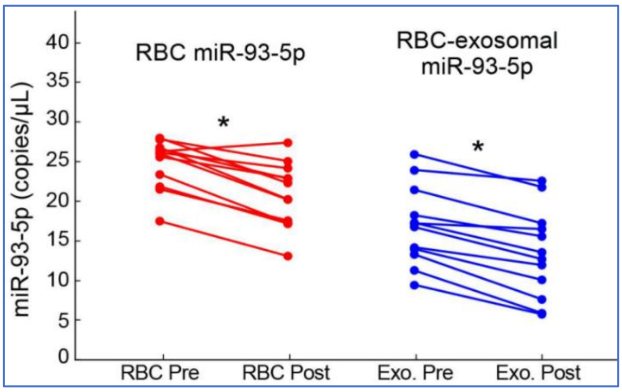

Figure S13. Paired pre- and post-surgery analysis of RBC and RBC-exosomal miR-93-5p levels in lung cancer patients.

RBC and RBC-derived exosomal miR-93-5p expression levels were quantified by ddPCR in 12 lung cancer patients before and after surgical tumor resection. Each line connects paired samples from the same patient. Both RBC and RBC-exosomal miR-93-5p levels significantly decreased following surgery (paired two-tailed Student's t-test, RBC: *, $p < 0.05$. Please note that paired samples were available from only 12 patients.

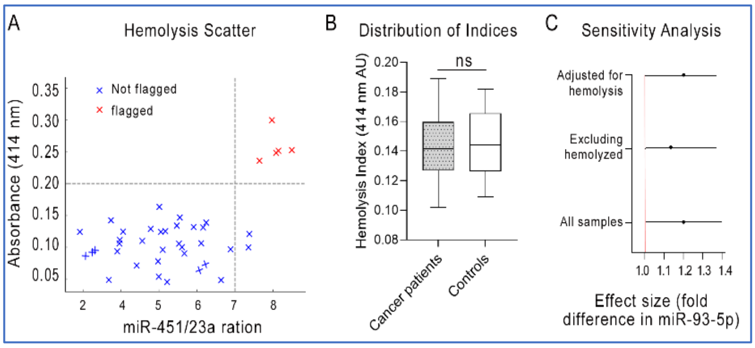

Figure S14. Hemolysis and sensitivity analyses for RBC-exosomal miR-93-5p.
(A) Scatterplot of hemolysis indices using the miR-451/23a ratio (x-axis) and absorbance at 414 nm (A414, y-axis). Five samples exceeding both thresholds were flagged as hemolyzed (red), while non-hemolyzed samples (35) are shown in blue. (B) Boxplots comparing A414 values between lung cancer patients and controls. Hemolysis markers, including A414, miR-451/23a ratio, and plasma haptoglobin, did not differ significantly between groups (all P > 0.05), confirming that elevated RBC-exosomal miR-93-5p in patients is not attributable to hemolysis. (C) Forest plot of effect sizes (fold difference in RBC-exosomal miR-93-5p between patients and controls) under three analytic approaches: all samples, after excluding hemolyzed samples, and after adjustment for hemolysis indices. Comparable results across these models demonstrate that the observed increase in miR-93-5p is robust and independent of hemolysis (P > 0.05).

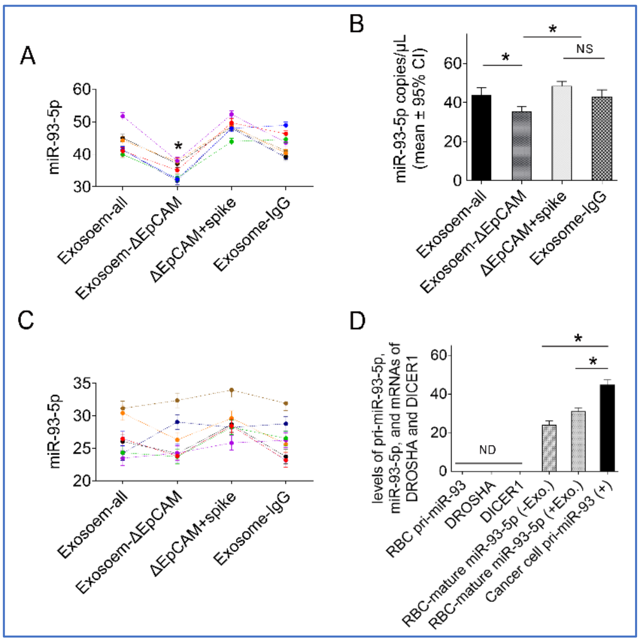

Figure S15. Tumor EpCAM⁺ exosomes transfer miR-93-5p into RBCs.
(A) In lung cancer patients, depletion of EpCAM⁺ exosomes (Exosome-ΔEpCAM, anti-EpCAM antibody) reduced RBC miR-93-5p levels compared with total exosomes (Exosome-all), while reconstitution with captured EpCAM⁺ exosomes (ΔEpCAM+Spike) restored the signal. Isotype control depletion (Exosome-IgG) showed no effect (n = 8 per condition). Statistical comparisons were performed using paired two-tailed t-tests. *P < 0.05. (B) Group summary of patient data (mean ± SD, copies/µL) confirmed the depletion–rescue pattern: Exosome-all, 47.5 ± 3.8; Exosome-ΔEpCAM, 42.7 ± 1.4; ΔEpCAM+Spike, 56.8 ± 2.0; Exosome-IgG, 52.1 ± 2.8. Exosome-ΔEpCAM was significantly lower than Exosome-all (*P < 0.05), and ΔEpCAM+Spike was significantly higher than Exosome-ΔEpCAM (*P < 0.05). Other comparison was not significant (NS). (C) Healthy controls exhibited lower baseline RBC miR-93-5p levels with minimal changes across exosome fractions, consistent with the absence of tumor-derived EpCAM⁺ exosomes (all p>0.05). (D) Quantification of miR-93-5p, pri-miR-93, and miRNA of DROSHA and DICER1 by ddPCR. The Y-axis indicates absolute RNA copy number (copies/µL) for each target. RBCs exhibited undetectable levels of pri-miR-93, DROSHA, and DICER1, confirming the absence of an endogenous miRNA synthesis machinery. Mature miR-93-5p levels in untreated RBCs (RBC mature miR-93-5p, −Exo.) were low, but significantly increased after incubation with tumor-derived exosomes (RBC mature miR-93-5p, +Exo.; P < 0.05), indicating exosome-mediated transfer of miR-93-5p into RBCs. Cancer cells showed high pri-miR-93 expression, consistent with active endogenous transcription and processing capacity. Data are presented as mean ± SD, and statistical significance was determined using one-way ANOVA with Tukey's post-hoc test (P < 0.05). ND, not detectable.

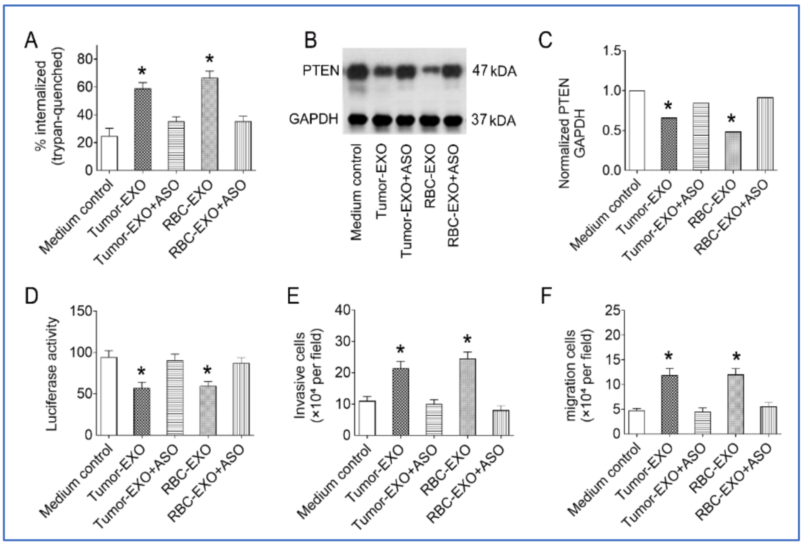

Figure S16. Head-to-head, equal-dose exosome delivery ± ASO-miR-93-5p in recipient PTEN-WT NSCLC cells.
(A) H460 PTEN-WT NSCLC cells were incubated with equal particle doses of tumor-derived exosomes (from A549 cells) or patient-derived RBC-exosomes. Exosome uptake was quantified by trypan blue quenching. Exposure to tumor- or RBC-derived exosomes increased attachment and internalization in recipient cancer cells (mean ± 95% CI; n = 5). (B) Western blot analysis of PTEN in H460 cell lysates after exosome exposure. Both tumor- and RBC-exosomes reduced PTEN protein abundance, which was rescued by ASO-miR-93-5p. (C) Densitometric quantification of PTEN normalized to GAPDH. Data is expressed relative to medium control. *$p < 0.05$ vs medium control; *$p < 0.05$ for ASO-miR-93-5p vs corresponding exosome-only treatment. (D) Luciferase reporter assays demonstrated suppression of PTEN-3' UTR activity by tumor- and RBC-exosomes (*$p < 0.05$ vs medium control). This repression was reversed by ASO-miR-93-5p (*$p < 0.05$ vs exosome-only). (E–F) Tumor- and RBC-exosomes enhanced invasion and migration of H460 cells (*$p < 0.05$ vs medium control). Both phenotypes were abrogated by ASO-miR-93-5p (*$p < 0.05$ vs exosome-only). Statistical analyses were performed using two-tailed Student's t-tests for pairwise comparisons and one-way ANOVA with post hoc Tukey tests for multiple group comparisons. Data are presented as mean ± 95% CI; ns, not significant.

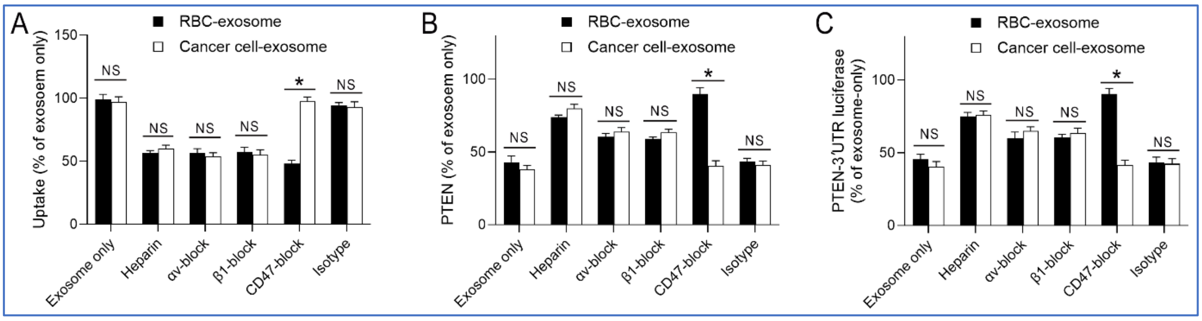

Figure S17. Surface-cue–dependent uptake distinguishes RBC-exosomal entry.
(A) Equal-dose uptake (% of exosome-only control) in H460 lung tumor recipient cells incubated with RBC-derived exosomes of lung cancer patients or tumor-derived exosomes from A549 cells. Uptake of exosomes by H460 recipient cells was measured in the presence of heparin, anti-integrin β1 antibody, anti-integrin αv antibody, anti-CD47 antibody, or isotype control. Heparin and integrin blockade reduced uptake of both RBC- and tumor-exosomes, whereas CD47 blockade selectively reduced uptake of RBC-exosomes. (B) PTEN protein rescue in H460 recipient cells after exosome treatment. Rescue of PTEN expression paralleled the uptake reduction, with CD47 blockade preferentially rescuing PTEN suppression by RBC-exosomes. (C) PTEN-3′UTR luciferase reporter activity in H460 recipient cells exposed to RBC-derived or A549 tumor-derived exosomes under the same inhibitory conditions as in panels (A) and (B). CD47 blockade selectively restored PTEN-3′UTR reporter activity that was otherwise suppressed by RBC-derived exosomes, confirming that uptake inhibition limits exosomes-mediated PTEN suppression. Data are presented as mean ± confidence interval (CI); n = 5 independent experiments, analyzed by one-way ANOVA with Tukey's post-hoc test.
